# Cancer-immune interactions in ER-positive breast cancers: PI3K pathway alterations and tumor-infiltrating lymphocytes

**DOI:** 10.1186/s13058-019-1176-2

**Published:** 2019-08-07

**Authors:** Marcelo Sobral-Leite, Izhar Salomon, Mark Opdam, Dinja T. Kruger, Karin J. Beelen, Vincent van der Noort, Ronald L. P. van Vlierberghe, Erik J. Blok, Daniele Giardiello, Joyce Sanders, Koen Van de Vijver, Hugo M. Horlings, Peter J. K. Kuppen, Sabine C. Linn, Marjanka K. Schmidt, Marleen Kok

**Affiliations:** 1 Division of Molecular Pathology, Netherlands Cancer Institute, Amsterdam, The Netherlands; 2 Coordenação de Pesquisa, Instituto Nacional do Câncer, Rio de Janeiro, RJ Brazil; 3 Division of Pathology, Netherlands Cancer Institute, Amsterdam, The Netherlands; 4 Department of Pathology, Netherlands Cancer Institute, Amsterdam, The Netherlands; 5 Department of Pathology, Ghent University Hospital, Ghent, Belgium; 6 Department of Medical Oncology, VU University Medical Centre, Amsterdam, The Netherlands; 7 Department of Biometrics, Netherlands Cancer Institute, Amsterdam, The Netherlands; 8 Division of Medical Oncology, Reinier de Graaf Hospital, Delft, The Netherlands; 9 Division of Medical Oncology, Netherlands Cancer Institute, Amsterdam, The Netherlands; 10 Department of Pathology, University Medical Center Utrecht, Utrecht, The Netherlands; 11 Department of Surgery, Leiden University Medical Center, Leiden, The Netherlands; 12 Department of Medical Oncology, Leiden University Medical Centre, Leiden, The Netherlands; 13 Division of Molecular Oncology and Immunology, Netherlands Cancer Institute, Plesmanlaan 121, 1066 CX Amsterdam, The Netherlands

**Keywords:** Luminal breast cancer, Tumor-infiltrating lymphocytes, *PIK3CA* mutations, PI3K pathway

## Abstract

**Introduction:**

The presence of tumor-infiltrating lymphocytes (TILs) is correlated with good prognosis and outcome after (immuno)therapy in triple-negative and HER2-positive breast cancer. However, the role of TILs in luminal breast cancer is less clear. Emerging evidence has now demonstrated that genetic aberrations in malignant cells influence the immune landscape of tumors. Phosphatidylinositol 3-kinase (PI3K) is the most common altered pathway in ER-positive breast cancer. It is unknown whether changes in the PI3K pathway result in a different composition of the breast tumor microenvironment. Here we present the retrospective analysis of a prospective randomized trial in ER-positive breast cancer on the prognostic and predictive value of specific tumor-associated lymphocytes in the context of PI3K alterations.

**Methods:**

We included 563 ER-positive tumors from a multicenter trial for stage I to III postmenopausal breast cancer patients, who were randomized to tamoxifen or no adjuvant therapy. The amount of CD8-, CD4-, and FOXP3-positive cells was evaluated by immunohistochemistry and quantified by imaging-analysis software. We analyzed the associations between *PIK3CA* hotspot mutations, PTEN expression, phosphorylated proteins of the PI3K and MAPK pathway (p-AKT, p-ERK1/2, p-4EBP1, p-p70S6K), and recurrence-free interval after adjuvant tamoxifen or no adjuvant treatment.

**Results:**

CD8-positive lymphocytes were significantly more abundant in *PIK3CA*-mutated tumors (OR = 1.65; 95% CI 1.03–2.68). While CD4 and FOXP3 were not significantly associated with prognosis, patients with tumors classified as CD8-high had increased risk of recurrence (HR = 1.98; 95% CI 1.14–3.41; multivariable model including *PIK3CA* status, treatment arm, and other standard clinicopathological variables). Lymphocytes were more often present in tumors with increased PI3K downstream phosphorylation. This was most pronounced for FOXP3-positive cells.

**Conclusion:**

These exploratory analyses of a prospective trial in luminal breast cancer suggest high CD8 infiltration is associated with unfavorable outcome and that PI3K pathway alterations might be associated with the composition of the tumor microenvironment.

**Electronic supplementary material:**

The online version of this article (10.1186/s13058-019-1176-2) contains supplementary material, which is available to authorized users.

## Introduction

The prognosis of early luminal breast cancer patients has substantially improved after the introduction of endocrine therapy (tamoxifen and aromatase inhibitors) [1]. Even so, around 20% of patients still experience recurrence of disease [2]. Accumulating evidence including multiple independent studies and meta-analyses have shown the correlation between tumor-infiltrating lymphocytes (TILs) and favorable prognosis in triple-negative breast cancer (TNBC) and human epidermal growth factor receptor 2 (HER2)-positive breast cancer [3, 4]. The prognostic value of TILs in ER-positive/HER2-negative disease remains relatively unclear, and conflicting results have been reported [3, 5–13]. There is no solid biological explanation for this different role of TILs in ER-positive/HER2-negative breast cancer. Recently, emerging experimental and clinical studies have demonstrated that genetic aberrations in cancer cells can influence the immune landscape of tumors [14–18]. Common drivers of tumorigenesis, such as but not limited to p53, Notch, and MYC, can modulate immune signaling pathways [14–18]. Studying the relationship between the genetic make-up of luminal breast carcinomas and the infiltration of specific subsets of immune cells is crucial for the rational design of clinical trials evaluating the combination of novel targeted therapy with immunomodulatory agents.

The phosphatidylinositol 3-kinase (PI3K) pathway is one of the most altered pathways in luminal breast cancer, and large efforts to unravel endocrine resistance have pointed to aberrant signaling of PI3K, e.g., by *PIK3CA* mutations, loss of PTEN, or downstream protein phosphorylation [19–23]. It led to the development of treatment strategies like mTOR and PI3K inhibitors [19, 21, 24, 25]. Adding PI3K inhibitors to standard endocrine treatment results in improvement of progression-free survival (PFS) in patients with metastatic disease with a *PIK3CA*-mutated tumor [25]. So far, it is largely unknown whether PI3K alterations influence the composition of the tumor microenvironment.

Another treatment strategy that is currently under investigation in luminal breast cancer includes immune checkpoint blockade. Although first results suggest that response rates are modest, durable responses have been observed in luminal metastatic breast cancer [26]. For further improvement of immunomodulatory treatments for luminal breast cancers, evaluating the presence of specific immune cell subsets as well as a better understanding of the cancer-immune interactions will be crucial.

T lymphocytes are involved in the cytotoxic adaptive immunity and play a central role in antitumor immune responses [27, 28]. Different subpopulations of T lymphocytes regulate the balance of immune tolerance and pro-inflammatory status in tumors [17, 28–30]: CD8-positive cytotoxic T lymphocytes are involved in interferon gamma (IFNγ)–dependent mechanisms of antitumor activity [11, 28, 29]; CD4-positive helper T lymphocytes are important in activating and regulating the adaptive immune response [28, 30]; in contrast, FOXP3-positive regulatory T lymphocytes have a crucial role in protecting tissues from inflammation damage and preventing autoimmunity [13, 30]. In the tumor microenvironment, regulatory T lymphocytes play key roles in inhibiting antitumor immune responses [29, 31].

A deeper understanding of the prognostic role of these TIL subsets, their predictive value for endocrine treatment benefit, and the relationship between the TIL subsets and PI3K activity will be useful for future trial design. It will help to find the optimal patient population for the evaluation of immunotherapy and/or PI3K inhibitors in ER-positive breast cancer. Here we present results of analyses on the prognostic and predictive value of specific tumor-associated lymphocytes in the context of PI3K alterations, using data from a trial in which breast cancer patients were randomized between adjuvant tamoxifen and no endocrine treatment.

## Materials and methods

### Study population and samples

The IKA trial recruited stage I to III postmenopausal breast cancer patients between 1982 and 1994. Patients were randomized (2:1) between 1 year of tamoxifen (30 mg per day) and no adjuvant therapy. After 1 year, a second randomization was performed between another 2 years of tamoxifen or to stop further treatment. None of the patients received chemotherapy. The study data were part of an Oxford systematic review that evaluated the effect of tamoxifen and were previously analyzed to study and validate biomarkers for tamoxifen resistance [1, 22, 23, 32–34]. Of 1662 patients included, we were able to retrieve 739 formalin-fixed paraffin-embedded (FFPE) tumor blocks. Tissue microarrays (TMAs) were constructed using three cores (0.6 mm each) per tumor. Of those tumors, 563 were ER-positive (ERα positivity defined as ≥ 10% positive tumor cells as determined using immunohistochemistry) [22, 23] (Additional file 1: Figure S1). Data collection of patient characteristics, including the pathology variables, expression of progesterone receptor (PR), HER2, and clinical outcome, was described previously [22, 23]. Ki67 staining was evaluated by the percentage of nuclear positive tumor cells (Additional file 2: Table S1). The original trial was approved by the central ethics committee of the Netherlands Cancer Institute (NKI), and informed consent was obtained from all study participants.

### *PIK3CA* mutation status

DNA isolated from FFPE tumor blocks was genotyped using Sequenom mass spectrometry–based genotyping technology to assess the hotspot mutations of the *PIK3CA* gene: in exon 9, 1624G>A (E542K) and 1633G>A (E545K), and exon 20, 3140A>T (H1047L) and 3140A>G (H1047R), as described previously [23]. Genotyping for *PIK3CA* exon 9 mutations was successful in 488 tumor samples, while exon 20 mutations could be assessed in 491 tumor samples [23] (Additional file 1: Figure S1).

### Evaluation of PI3K pathway activity

Phosphorylation status of proteins from the PI3K pathway: p-AKT (Thr308 and 473), p-4EBP1 (Ser65), and p-p70S6K (Thr389); from the MAPK pathway: p-ERK1/2 (Thr202/Tyr204); and PTEN expression were assessed using immunohistochemistry (IHC) on TMAs. The details on the IHC analyses and the criteria used for scoring of these markers were previously reported and are summarized in Additional file 2: Table S1 [22, 23, 33, 34].

### Evaluation of the lymphocyte markers

Whole serial sections of 563 ER-positive FFPE tissue blocks were stained for CD4, CD8, and FOXP3. IHC was performed using the Ventana Benchmark® Ultra system (Ventana Medical Systems, Tucson, USA), and details are provided in Additional file 2: Table S1. The slides stained for CD8 and CD4 were scanned using the Aperio Scanner (Leica Biosystems, San Diego, CA, USA) and automatically evaluated by image-analysis software (Tissue Image Analysis, version 2.0, Slidepath, Leica Biosystems, Milton Keynes, UK). The Slidepath software was manually trained to identify and quantify cells in the image of the scanned tumor sections according to staining color, size, and shape of the cells, similar as previously reported [35]. Based on this data, the software calculated the percentage of positive CD4- and CD8-positive lymphocytes among all cells of the tumor tissue section. Slides stained for FOXP3 were scanned using the Philips Ultra Fast Scanner 1.6 RA. FOXP3 nuclear positivity of lymphocytes in intratumoral and in surrounding stroma tissue was automated measured by AxioVision 4.6 (Carl Zeiss Vision, Jena, Germany), as previously described [8]: four fields per slide (in average 1.5 mm^2^ per field) were used to count the number of FOXP3-positive cells per mm^2^.

Since the distribution of the expression values of these markers showed lognormal properties, we took the logarithm of these values and centered the resulting distributions in zero by subtracting the logarithmic median of each marker. Two certified pathologists (HH and KVdV) and a molecular biologist (Iz) scored the percentage of CD4-, CD8-, and FOXP3-positive lymphocytes in a selected number of cases (HH: *n* = 33, KVdV: *n* = 30, and Iz: *n* = 100). Samples quantified as zero in the automated and normalized expression correspond, approximately, to the following pathologists’ scores: 5% CD4-, 15% CD8-, and 1% FOXP3-positive cells, respectively (Additional file 1: Figure S2). From the 563 ER-positive breast cancer patients included in the trial, 161 to 185 tumor samples had insufficient tumor material to evaluate the expression of these markers. We could quantify CD4, CD8, and FOXP3 expression in 396, 402, and 378 tumor samples, respectively (Additional file 1: Figure S1).

### Statistical analyses

Associations were evaluated by the chi-square test or by Fisher exact test if one of the expected counts were less than 5. Comparison between pathologists’ and the expression of lymphocyte markers obtained by automated fashion was calculated by Spearman’s rank correlation test and by the intraclass correlation coefficient (ICC; assuming two-way random single measures) [36]. Comparisons of means between two or more groups were examined by Student’s *t* test or ANOVA, respectively. Odds ratios (OR) with their respective 95% confidence intervals (95% CI) were calculated using binomial logistic multivariable regression models to measure the association between lymphocyte markers and *PIK3CA* mutation status. Associations between tumor variables and lymphocytes or (phospho-) protein levels were calculated by linear regression models and quantified in regression coefficients (*r*) and their 95% CI.

Survival time was calculated from the date of randomization. End of follow-up was defined as the date of an event, lost to follow-up, or 10 years time at risk (censoring), whatever came first. The recurrence-free interval (RFI) was defined as the time from the date of first randomization until the occurrence of a local, regional, or distant recurrence, or breast cancer-specific death [22]. Breast cancer-specific interval (BCSI) was defined as death caused by breast cancer progression or a breast cancer treatment-related event. Secondary primary breast tumor was not considered an event, and these patients were censored at the date of this occurrence [22].

Hazard ratios (HRs) and 95% CI were estimated using Cox regression models. Of note, after 1989, two interim analyses showed a significant improvement in RFI in lymph node (LN)-positive patients. After this year, LN-positive patients in this trial skipped the first randomization and all received 1 year of tamoxifen [32]. Therefore, multivariable Cox regression models were stratified by LN status. Regression analysis included the following variables in the (logistic, linear, and Cox) multivariable models: morphology type (ductal, lobular, or others), tumor grade (1, 2, or 3), tumor size (≤ 2 cm or > 2 cm), PR (negative or positive), HER2 (negative or positive), and *PIK3CA* mutation status (wild-type or mutated). Cox regression models also included age at diagnosis (as continuous value) and tamoxifen arm (treated or control). Splined curves of the Cox regression terms against the expression of lymphocyte markers were used to check for non-linear associations (degree of freedom = 3) [37]. Cut-off estimations for the lymphocyte markers (low or high) were based on the best sensitivity and specificity measures for RFI prediction [38]. We used a freely available web application to calculate the threshold (R package: *Optimal.cutpoints*). The method (*MaxKappa*) makes full use of the clinical and molecular information to assess the prediction value over chance [39, 40].

Unsupervised hierarchical clustering and Euclidean dissimilarity was conducted on phosphorylation levels and protein expression values, centered and proportionally scaled. Results of regression models were obtained using patients with complete data only.

All *p* values reported are from two-sided tests, and the threshold for significance was set at *p* = 0.05. No correction for multiple testing was applied. All statistical analyses were performed using RStudio version 3.2.3 (RStudio Team, 2015).

## Results

### CD4, CD8, and FOXP3 expression and standard clinicopathological features

**Fig. 1:**
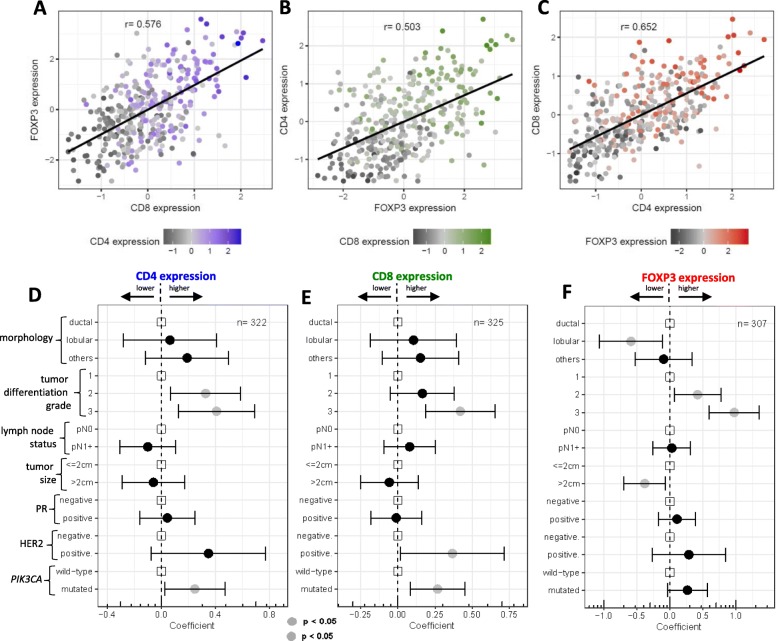
Correlations between the expression of lymphocyte markers and pathological features. **a**–**c** Correlation plots of the expression of the three lymphocyte markers. Each graph includes a regression line and the Spearman coefficient of the correlation between the two markers; the expression of the third marker is indicated by the color degree of the dots: CD8 in blue, CD4 in green, and FOXP3 in red. **d**–**f** The plots graphically present the magnitude of the association between each pathological feature and the expression of lymphocyte markers: CD4 (**d**), CD8 (**e**), and FOXP3 (**f**). Coefficients with 95% confidence intervals were estimated using multivariable linear regression models (based on cases with complete information). Abbreviations: pN0 lymph node negative, pN1+ lymph node positive, PR progesterone receptor, HER2 human epidermal growth factor receptor 2, *PIK3CA* mutations in exon 9 and/or exon 20

Cells positively stained for CD4, CD8, and FOXP3 were mainly found in the stromal areas (Additional file 1: Figure S3A-F). The average scores from two pathologists were compared with the expression values obtained by image-analysis software (*r*_CD4_ = 0.752, *r*_CD8_ = 0.796, and *r*_FOXP3_ = 0.836; Additional file 1: Figure S3G-I). The presence of CD4-, CD8-, and FOXP3-positive cells was strongly correlated (*r*_CD8/FOXP3_ = 0.576, *r*_FOXP3/CD4_ = 0.503, and *r*_CD4/CD8_ = 0.652; Fig. 1a–c).

Figure 1d–f shows the correlation between CD4-, CD8-, and FOXP3-positive cells and clinicopathological characteristics as calculated by multivariable linear regression. More CD8-, CD4-, and FOXP3-positive cells were detected in high-grade tumors (grade 3) (all *p* < 0.001; univariable analyses in Additional file 1: Figure S4A-C). In line with these associations, tumors with high Ki67 scores were associated with higher levels of CD4, CD8, and FOXP3 (*p* < 0.001; Additional file 1: Figure S4D-F). HER2-positive tumors showed higher CD8 expression compared with HER2-negative tumors (*r*_CD8_ = 0.36, *p* = 0.040; Fig. 1e). FOXP3 expression was significantly lower in large tumors (> 2 cm; *r*_FOXP3_ = − 0.38, *p* = 0.018) and in lobular carcinomas (*r*_FOXP3_ = − 0.59, *p* = 0.015; Fig. 1f), compared with ductal carcinomas (*p* < 0.001; Additional file 1: Figure S4I).

### CD4, CD8, and FOXP3 expression and *PIK3CA* mutations

**Fig. 2:**
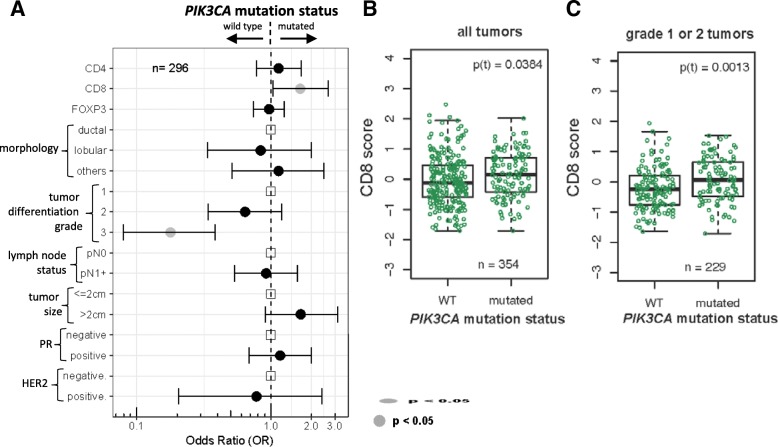
Expression of the lymphocyte markers and *PIK3CA* mutation status. **a** Forest plot graphically represents the magnitude of the association between each pathological feature and *PIK3CA* mutation status, calculated by multivariable logistic regression model (as complete case analysis). **b** Distribution of CD8 expression according to *PIK3CA* mutation status in all tumors and **c** within grade 1 or 2 tumors. Statistical differences between the expression means among the two categories were calculated by *t* test: p(t). Abbreviation: pN0 lymph node negative, pN1+ lymph node positive, PR progesterone receptor

*PIK3CA* mutations in exon 9 or exon 20 were found in 159 (32.7%) of the 486 tumors genotyped [23]. In total, 296 patients had complete information for *PIK3CA* mutation and the three immune markers. The tumor microenvironment of *PIK3CA*-mutated tumors was infiltrated with relatively more CD8-positive cells compared to wild-type tumors (*p* = 0.038, Fig. 2a, b). Linear multivariable regression models showed a relatively weak but statistically significant correlation between *PIK3CA* mutation status and higher CD4 and CD8 levels (*r*_CD4_ = 0.25, *p* = 0.030 and *r*_CD8_ = 0.27, *p* = 0.005; Fig. 1d, e).

Two specific *PIK3CA* mutations were associated with CD8 infiltration: tumors harboring 1633G>A mutations in exon 9 or 3140A>G in exon 20 showed slightly higher CD8 expression compared with the wild-type tumors (*p*_1633G>A_ = 0.0473; Additional file 1: Figure S5B and *p*_3140A>G_ = 0.0322; Additional file 1: Figure S5E).

As previously reported by our group and others [23, 41], low-grade tumors (grade 1 or 2) are likely to have more *PIK3CA* mutations (OR_grade 3vs1_ = 0.18; 95% CI 0.08–0.38; Fig. 2a). Within these low-grade tumors, the correlation between CD8 levels and *PIK3CA* mutation status was more pronounced (*p* = 0.001; Fig. 2c).

### CD4, CD8, and FOXP3 expression and breast cancer outcome

**Fig. 3:**
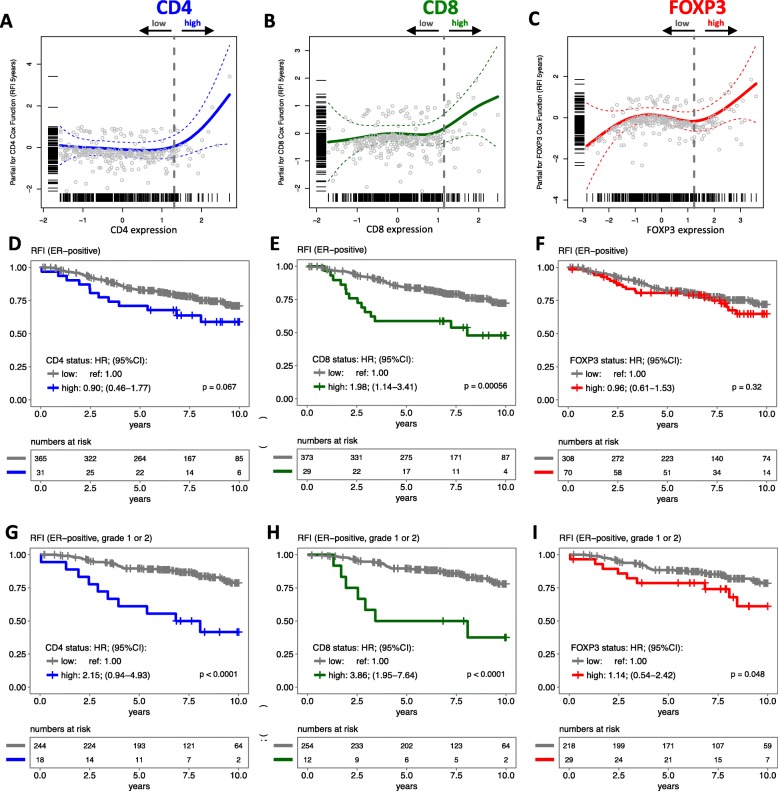
Association between CD4, CD8, and FOXP3 expression and relapse-free interval (RFI). Partial residuals of the multivariable Cox regression models were plotted against the CD4 (**a**), CD8 (**b**), and FOXP3 (**c**) expression. Smoothed lines were drawn based on the spline function of the fitted partial residual values of the Cox regression (RFI; 5 years follow-up) and their standard error range. Vertical gray dashed lines show the threshold used to classify status low and high for each lymphocyte marker, estimated by sensitivity/specificity measurements of differences in RFI. Kaplan-Meier curves and adjusted hazard ratios (HRs) of CD4, CD8, and FOXP3 status were calculated in the whole group (**d**, **e**, and **f**, respectively) and among patients with low-grade tumors (**g**, **h**, and **i**, respectively). Statistical differences between the groups were calculated by the log-rank test (*p*). Multivariable models were stratified by lymph node status and included the following variables: CD4, CD8, and FOXP3 status; morphology type; tumor grade; tamoxifen arm; tumor size; age at diagnosis; PR; HER2; and *PIK3CA* mutation status. Abbreviation: HR hazard ratio, CI confidence interval, ER estrogen receptor, RFI relapse-free interval

Continuous CD4, CD8, and FOXP3 expression levels were not significantly associated with RFI in the Cox linear regression models (data not shown). To illustrate this, we plotted the partial residuals of the (univariate and multivariable) Cox regression models for each lymphocyte marker (Additional file 1: Figure S6A-F). However, it is known that continuous variables can be associated with the hazard in a non-linear way [42]. The spline function of the fitted partial residual values of the Cox regression models for each lymphocyte marker indeed showed a non-linear shape (Additional file 1: Figure S6G-I). Since there is no established cut-off for CD4, CD8, and FOXP3 available yet, these spline plots were used to identify the best cut-off points in the scale of the expression of the lymphocyte markers according to the largest differences in the recurrence rates (Additional file 1: Figure S6J-L). The splined curves highlighted the increase of partial residual values of patients classified as high CD4, CD8, and FOXP3 (Fig. 3a–c). The Kaplan-Meier curves illustrate that patients with tumors harboring high levels of CD8 (29 of the total of 410 tumors) have a worse RFI compared to patients with low CD8 tumors (log-rank test; *p* < 0.001; Fig. 3e). After multivariable adjustment for *PIK3CA* mutation and other pathological variables, this association remained significant: HR_high-CD8_ = 1.98; 95% CI 1.14–3.41 (Fig. 3e and Additional file 1: Figure S7A). Associations between RFI and CD4 and FOXP3 expression were not statistically significant after multivariable analysis (Fig. 3d, f, and Additional file 1: Figure S7A). After exclusion of the patients with HER2-positive disease, we still observe a significantly worse RFI in patients with high CD8 expression in the tumor microenvironment (HR_high-CD8_ = 1.87; 95% CI 1.07–3.28, *p* < 0.0001).

While ER-positive breast tumors with low grade are considered to have a relative good prognosis in breast cancer [1], within these low-grade tumors, we observed that high levels of CD4 or CD8 were associated with poor outcome (log-rank test; *p* < 0.0001; Fig. 3g, h). The effect remained significant after adjustment for known prognostic factors (Fig. 3g, h, and Additional file 1: Figure S7B). Similar associations were observed when using BCSI as endpoint (Additional file 1: Figure S7C and D).

### CD8 expression and breast cancer outcome according to tamoxifen treatment

**Fig. 4:**
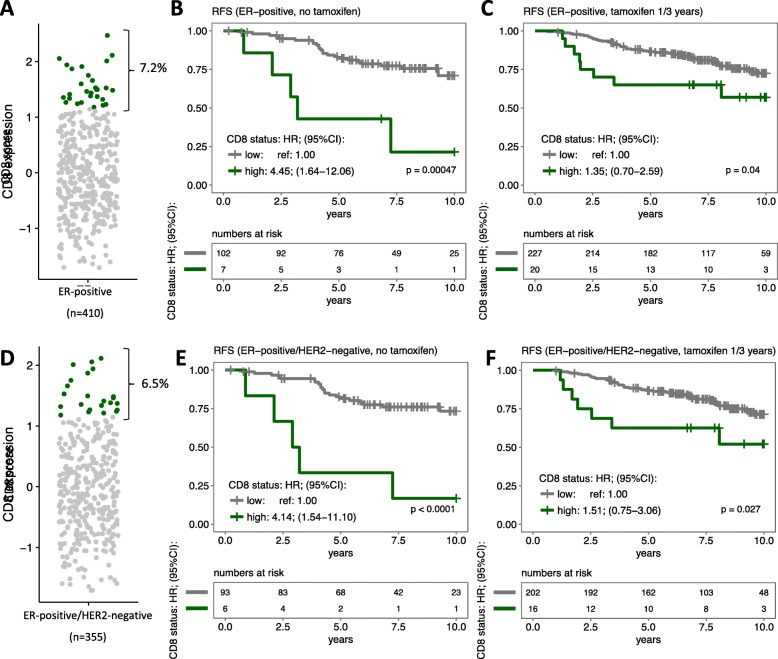
Association between CD8 status and tamoxifen benefit. The boxplots show the distribution of CD8 scores among all ER-positive tumors (**a**). The proportion of patients with the tumors classified as high-CD8 based on the cut-off defined in Fig. 3 are plotted in green. Kaplan-Meier curves and adjusted hazard ratios (HRs) of CD8 status were calculated in patients who did not receive adjuvant tamoxifen (**b**) and who received adjuvant tamoxifen for 1 or 3 years (**c**). The same analysis was applied within ER-positive/HER2-negative patients (**d**, **e**, and **f**). Statistical differences in survival were calculated by the log-rank test (*p*). Multivariable models were stratified by lymph node status and included CD4, CD8, and FOXP3 status; morphology type; tumor grade; tumor size; age at diagnosis; PR; HER2 (when applicable); and *PIK3CA* mutation status. Abbreviation: HR hazard ratio, CI confidence interval, ER estrogen receptor, RFS relapse-free survival

The proportion of patients identified with CD8-high tumors corresponded to only 7.2% of total tumors quantified (*n* = 410; Fig. 4a). We evaluated the prognostic value of CD8 status within each arm of the trial: not-treated and treated with tamoxifen (1 or 3 years after surgery). High CD8 levels were associated with poor RFI regardless of tamoxifen treatment (log-rank test; *p* < 0.001 and *p* = 0.040, respectively; Fig. 4b, c). However, univariate HR remained significant only for the control arms. The *p* value for interaction between CD8 status and tamoxifen treatment in a full model for RFI was 0.082. Since patients with HER2-positive tumors did not receive trastuzumab, we applied the same analysis in the HER2-negative patients only. The proportion of CD8-high tumors and RFI associations observed in this subgroup (*n* = 355) were similar to whole ER-positive group.

Of note, after the first interim analyses of the IKA trial, it was decided that LN-positive patients would skip the first randomization. After this point, all LN-positive patients received 1 year of tamoxifen [32]. Thus, we calculated the effect of adjuvant tamoxifen during 1 or 3 years according to CD8 status, by applying regression analysis grouped by LN status, but it did not change our conclusions (Additional file 1: Figures S8, S9, and Additional file 2: Table S2).

### CD4, CD8, and FOXP3 expression and the activation of PI3K pathway in breast tumors

**Fig. 5:**
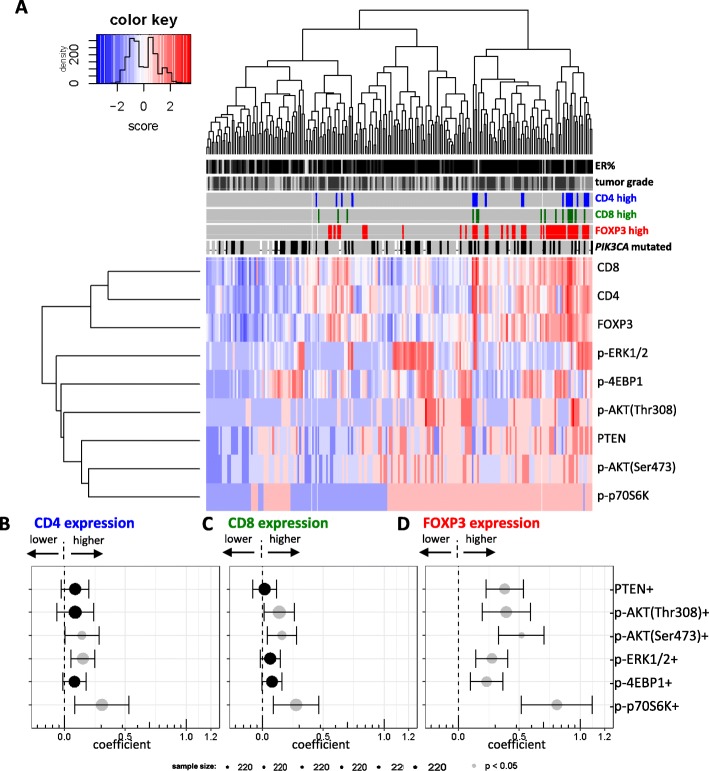
Lymphocytic infiltration and PI3K pathway activation in breast tumors. **a** Heat map represents unsupervised hierarchical clustering of 215 breast carcinomas (columns) based on the expression of lymphocyte markers and the activation of PI3K pathway on tumor cells (rows). Information on percentage of ER positivity (from 10 to 100%) and tumor grade (1, 2, or 3) is indicated by gray scale: low as lighter gray and high as darker gray. Boxes of *PIK3CA* mutation status are filled with gray (wild-type), black (mutated), or white (unknown). CD4, CD8, and FOXP3 status was filled with color (high) or gray (low). The forest plots represent multivariable linear regression models. Each plot merges 6 multivariable models (described in Additional file 2: Tables S2A-F) calculating the association between the (phospho-) levels of PI3K downstream proteins and CD4 (**b**), CD8 (**c**), or FOXP3 (**d**) expression. Measurements of these associations are represented by the coefficients plotted on the *x* axes. The size of each dot is proportional to the number of samples used for each multivariable complete case analysis, and bars represent the standard deviation

Unsupervised hierarchical clustering revealed that tumors with increased levels of CD4, CD8, and FOXP3 also had higher phosphorylation levels of proteins from the PI3K pathway (right side of dendrogram and heat map, Fig. 5a).

In order to statistically evaluate this link between the specific phosphorylation of the PI3K pathway and the presence of lymphocytes, we performed multivariable linear regression models including pathological variables and *PIK3CA* mutation status (Additional file 2: Tables S3A-F). In general, we found a weak positive correlation between the phosphorylation levels of PI3K pathway and lymphocyte infiltration. More specifically, p-AKT(Ser473), p-ERK1/2, and p-p70S6K were statistically significant associated with high CD4 (*p* = 0.045, 0.003, and 0.007, respectively; Fig. 5b). A similar pattern was observed for the associations with CD8, in which p-AKT(Thr308), p-AKT(Ser473), and p-p70S6K reached statistical significance (*p* = 0.030, 0.009, and 0.004, respectively; Fig. 5c). Phosphorylation levels of the PI3K markers and PTEN expression were all positively and significantly correlated with FOXP3 expression (almost all *p* < 0.001, Fig. 5d and Additional file 2: Tables S3A-F). Exclusion of the HER2-positive cases did not substantially change the results (data not shown).

Taking together, ER-positive breast tumors with high levels of TILs show more activation of downstream proteins of the PI3K pathway.

## Discussion

We performed post hoc analysis of TIL composition and PI3K alterations in a trial that compared the outcome of ER-positive breast cancer patients, randomized between adjuvant tamoxifen (1–3 years), and patients who received no adjuvant treatment. For CD4 and FOXP3 expression, we did not find an association with outcome. In contrast, our analyses in the total cohort of ER-positive tumors as well as in the ER-positive/HER2-negative subgroup suggest that patients with ER-positive tumors with very high infiltration of CD8-positive lymphocytes are more likely to have recurrence of disease. This association is in the opposite direction of the established link observed between high TILs and improved prognosis in TN and HER2-positive breast cancer [4, 5, 7, 11]. But in the largest observational study so far on CD8, Ali et al. described a relatively poor outcome for CD8-high tumors within the ER-positive/HER2-negative subgroup [5]. In addition, our exploratory data is also in line with a recent meta-analysis showing that an increase in TILs is associated with shorter overall survival in luminal–HER2-negative tumors (HR = 1.10; 95% CI 1.02–1.19, *p* = 0.011) [7]. Even though we determined CD8-high threshold using the survival data of this series, which resulted in overfitting, these large studies support the link we found between high CD8 expression and poor survival in luminal breast cancer. A clear explanation for this opposite effect of TIL in ER-positive disease versus patterns seen in TN or HER2-positive breast cancer is still lacking. We checked if tumors with high levels of CD8-positive lymphocytes would also be the ones expressing a lower percentage of ER positivity but no association was found (Additional file 1: Figure S10).

FOXP3 expression did not show significant prognostic value, similar to results from Ali et al. [5], Mahmoud et al. [12], and Baker et al. [6]. However, other studies have reported statistically significant associations between FOXP3+ and poor survival in breast cancer [9, 13]. Although FOXP3 status alone was not predictive for recurrence risk in our data, its expression was highly correlated with CD4 and CD8 expression. However, scoring of FOXP3-positive lymphocytes remains challenging for pathologists since these cells are often scattered in the stroma. Moreover, the dynamic range of FOXP3 expression in the breast tumor microenvironment is limited (0–15%; illustrated in Additional file 1: Figure S3I). Of note, we also analyzed the CD8/FOXP3 as well as the CD4/FOXP3 ratio and no significant association with survival was observed (data not shown). Further validation of the prognostic value of TIL subsets in luminal breast cancer is required.

The activation of the PI3K pathway plays a central role in cancer growth and tamoxifen resistance [19–21, 23]. Preclinical data suggested that deregulation of the PI3K pathway could contribute to immune escape [43]. On the other hand, detailed information on the cross-talk between the immune infiltrate and cancer cells harboring an aberration in the PI3K pathway in vivo is scarce. Using a large set of breast cancer patients who received adjuvant chemotherapy, Kotoula et al. did not show a clear association between TILs and *PIK3CA* mutations [44]. In order to investigate the association between the actual activation of the PI3K pathway and immune infiltration, we assessed phosphorylation levels of proteins downstream of PI3K in parallel with TIL subsets defined by CD4, CD8, and FOXP3 [22, 23]. Our analyses of TIL subsets in the context of PI3K alterations revealed that tumors with a *PIK3CA* mutation tend to have more CD8 cells and tumors enriched for FOXP3-positive cells show downstream activation of the PI3K pathway. Previously, we have shown the lack of clear association between mutations in *PIK3CA* and activation of downstream proteins in the PI3K/AKT/mTOR pathway [23]. *PIK3CA* exon 20 mutations are associated with higher p-ERK1/2 levels that belong to the MAP kinases pathway, and tumors with a *PIK3CA* exon 9 mutation are associated with higher p-AKT and p-ERK1/2, but not with p-p70S6K [23]. Our current study illustrates that cancer-immune interactions might differ depending on specific alterations in PI3K pathway. Interestingly, our data are in line with Crane et al., who demonstrated using in vitro models that breast carcinoma cells with activated PI3K pathway, once exposed to activated T cells, adopt an immune-resistant phenotype by increasing the percentage of FOXP3-expressing lymphocytes [43]. More functional studies are needed for a better understanding of the specific interactions between the multifunctional PI3K pathway and the various components of the pro- and anticancer immune response.

We also evaluated the benefit of adjuvant tamoxifen according to the CD8 status of their tumor. Within patients classified as CD8-low, no significant RFI improvement was detected when patients were treated with adjuvant tamoxifen, compared with the control group. Similarly, Blok et al. also observed a limited benefit of adjuvant tamoxifen relative to exemestane within patients with a low number of CD8 TILs [45]. More studies exploring the predictive value of tumor-immune profiles are needed.

Limitations of our study include the relatively small sample size and lack of correction for multiple testing. Furthermore, the patients included in our study did not receive endocrine treatment during 5–10 years or aromatase inhibitors, according to the current guidelines. Therefore, it remains to be determined whether tumor-associated immune cells are associated with long-term benefit of endocrine treatment. Although we evaluated the *PIK3CA*-immune interaction in a dataset of a prospective trial, this analysis was not preplanned and should therefore be considered exploratory.

Insufficient data is available on combination treatment with immunotherapy plus PI3K inhibition in luminal breast cancer. Recently, the randomized, phase 3 SOLAR-1 trial showed that the treatment with alpelisib, a PI3Kα-specific inhibitor, in combination with fulvestrant prolonged progression-free survival among patients with PIK3CA-mutated, ER-positive/HER2-negative advanced breast cancer [46]. Given that patients with a *PIK3CA* mutation in the tumor might have more benefit from PI3K inhibition [24, 25, 47], and, according to our data, could have more tumor-associated CD8, a combination treatment with immune checkpoint blockade plus PI3K inhibition deserves exploration in a clinical trial. Notably, combined PI3K and CDK4/6 inhibition, along with immune checkpoint inhibition induced durable regressions of TN breast cancer tumors in vivo [48]. Based on our observation that PI3K activation is associated with higher levels of regulatory T cells, the development of PI3K inhibitors in combination with immunomodulatory agents that are able to deplete regulatory T cells might be of interest for luminal breast cancer [43].

## Conclusion

In this dataset of ER-positive breast cancer, tumor infiltration of CD8-positive lymphocytes was associated with *PIK3CA* mutations and worse clinical outcome. These associations were more pronounced among patients with grade 1 or 2 tumors. Furthermore, activation of the PI3K pathway, measured using phosphorylated downstream proteins, in breast tumor cells was positively correlated with tumor-infiltrating FOXP3-positive lymphocytes. Further validation of these cancer-immune interactions in ER-positive breast tumors might provide useful information for further development of immunomodulatory combination treatments for ER-positive breast cancers with high TILs.

## Additional files

Additional file 1: **Figure S1.** Data collection. Source of data and tumor material from the patients entered in the multicenter IKA trial. **Figure S2.** Validation of the expression values generated by automated fashion. Panel of the comparison between expression evaluated by observers versus expression values obtained from image-analysis software (after normalization). **Figure S3.** Evaluation of the staining of the lymphocyte markers CD4, CD8 and FOXP3. **Figure S4.** Distribution of the expression of the lymphocyte markers by tumor characteristics. **Figure S5.** Distribution of the expression of lymphocyte markers according to *PIK3CA* mutation status. **Figure S6.** Analysis of the linearity of the Cox regression functions. **Figure S7.** Multivariable Cox regression models in ER-positive breast cancer. **Figure S8.** Association between CD8 status and tamoxifen benefit. **Figure S9.** Association between CD8 status and tamoxifen benefit within the HER2-negative group. **Figure S10.** Levels of the percentage of ER positivity are not associated with the status of lymphocyte markers. (PPTX 20084 kb)

Additional file 2: **Table S1.** Staining details. **Table S2.** Interaction terms. **Table S3.** PI3K pathway activation and lymphocytic infiltration. **Table S4.** Comparison between all patients and those with CD4, CD8, or FOXP3 staining. (PPTX 272 kb)

## Data Availability

Data from IKA trial (pathological, clinical, *PIK3CA* mutation status and PI3K phosphorylation pathways) were discussed in previous publications of the group in Breast Cancer Research [22, 23]. Data is available upon request from the corresponding author.
