# Supplementary material for: Cancer-immune interactions in ER-positive breast cancers: PI3K pathway alterations and tumor-infiltrating lymphocytes
Source: Breast Cancer Res. 2019 Aug 7;21:90. doi: 10.1186/s13058-019-1176-2 (PMC6686400; doi:10.1186/s13058-019-1176-2)
Supplement: Supplementary file 1 — Figure S1. Data collection. Source of data and tumor material from the patients entered in the multicenter IKA trial. Figure S2. Validation of the expression values generated by automated fashion. Panel of the comparison between expression evaluated by observers versus expression values obtained from image-analysis software (after normalization). Figure S3. Evaluation of the staining of the lymphocyte markers CD4, CD8 and FOXP3. Figure S4. Distribution of the expression of the lymphocyte markers by tumor characteristics. Figure S5. Distribution of the expression of lymphocyte markers according to PIK3CA mutation status. Figure S6. Analysis of the linearity of the Cox regression functions. Figure S7. Multivariable Cox regression models in ER-positive breast cancer. Figure S8. Association between CD8 status and tamoxifen benefit. Figure S9. Association between CD8 status and tamoxifen benefit within the HER2-negative group. Figure S10. Levels of the percentage of ER positivity are not associated with the status of lymphocyte markers. (PPTX 20084 kb) [file 13058_2019_1176_MOESM1_ESM.pptx]

## Slide 1
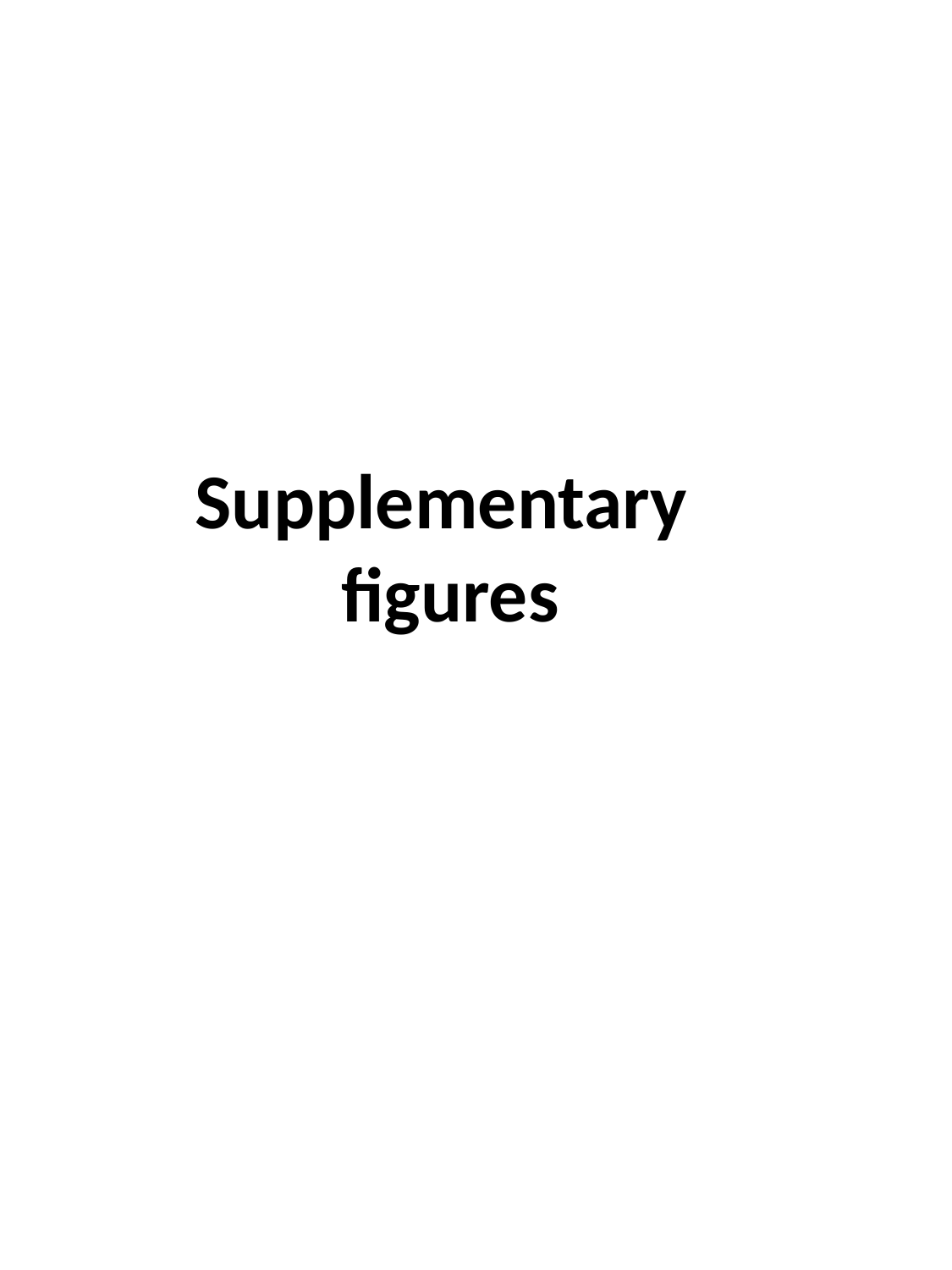

Supplementary
figures

## Slide 2
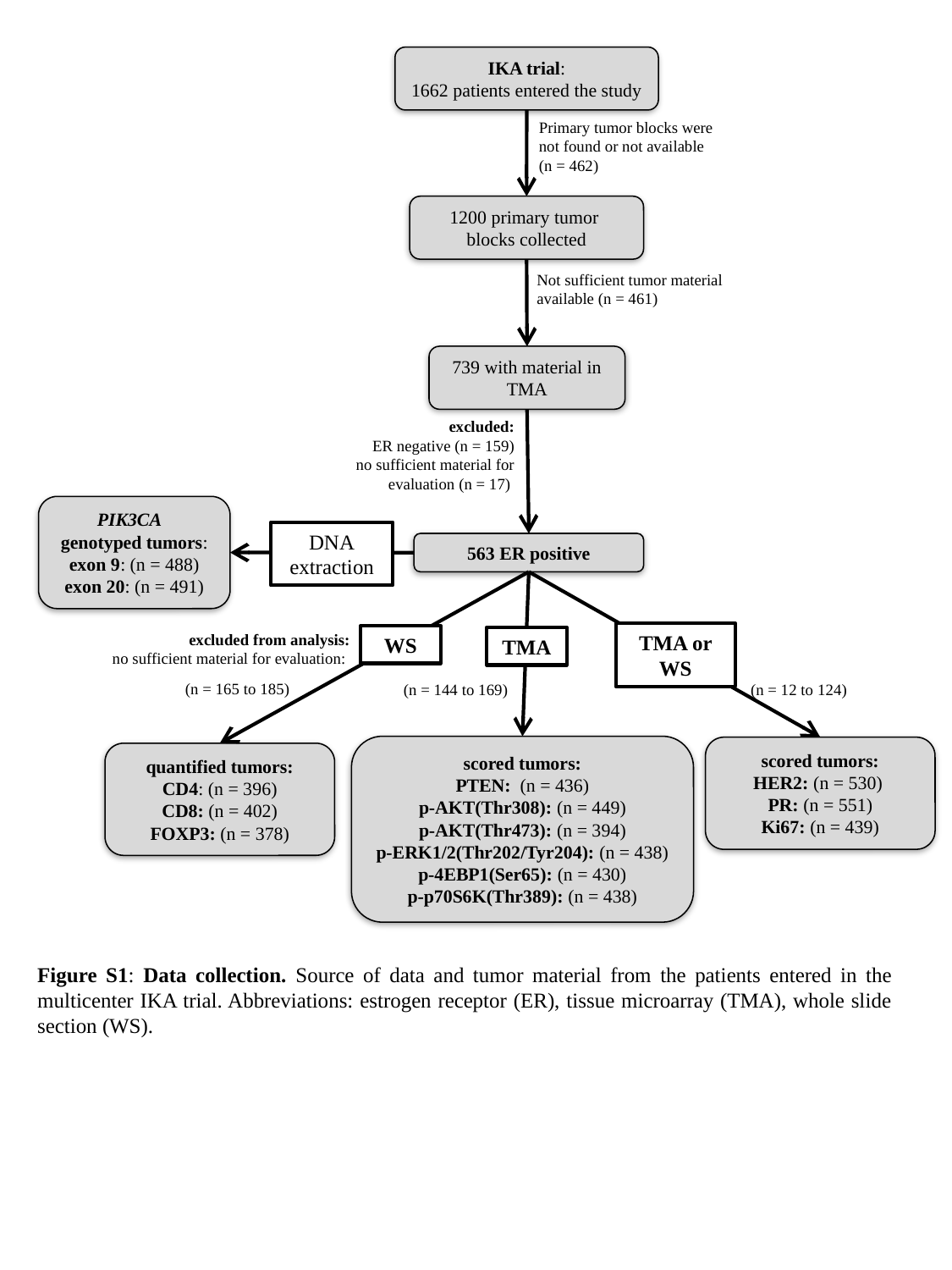

IKA trial:
1662 patients entered the study
Primary tumor blocks were not found or not available
(n = 462)
1200 primary tumor
blocks collected
Not sufficient tumor material available (n = 461)
739 with material in TMA
excluded:
ER negative (n = 159)
no sufficient material for evaluation (n = 17)
PIK3CA
genotyped tumors:
exon 9: (n = 488)
exon 20: (n = 491)
DNA
extraction
563 ER positive
excluded from analysis:
no sufficient material for evaluation:
TMA or WS
WS
TMA
(n = 165 to 185)
(n = 144 to 169)
(n = 12 to 124)
scored tumors:
PTEN: (n = 436)
p-AKT(Thr308): (n = 449)
p-AKT(Thr473): (n = 394)
p-ERK1/2(Thr202/Tyr204): (n = 438)
p-4EBP1(Ser65): (n = 430)
p-p70S6K(Thr389): (n = 438)
scored tumors:
HER2: (n = 530)
PR: (n = 551)
Ki67: (n = 439)
quantified tumors:
CD4: (n = 396)
CD8: (n = 402)
FOXP3: (n = 378)
Figure S1: Data collection. Source of data and tumor material from the patients entered in the multicenter IKA trial. Abbreviations: estrogen receptor (ER), tissue microarray (TMA), whole slide section (WS).

## Slide 3
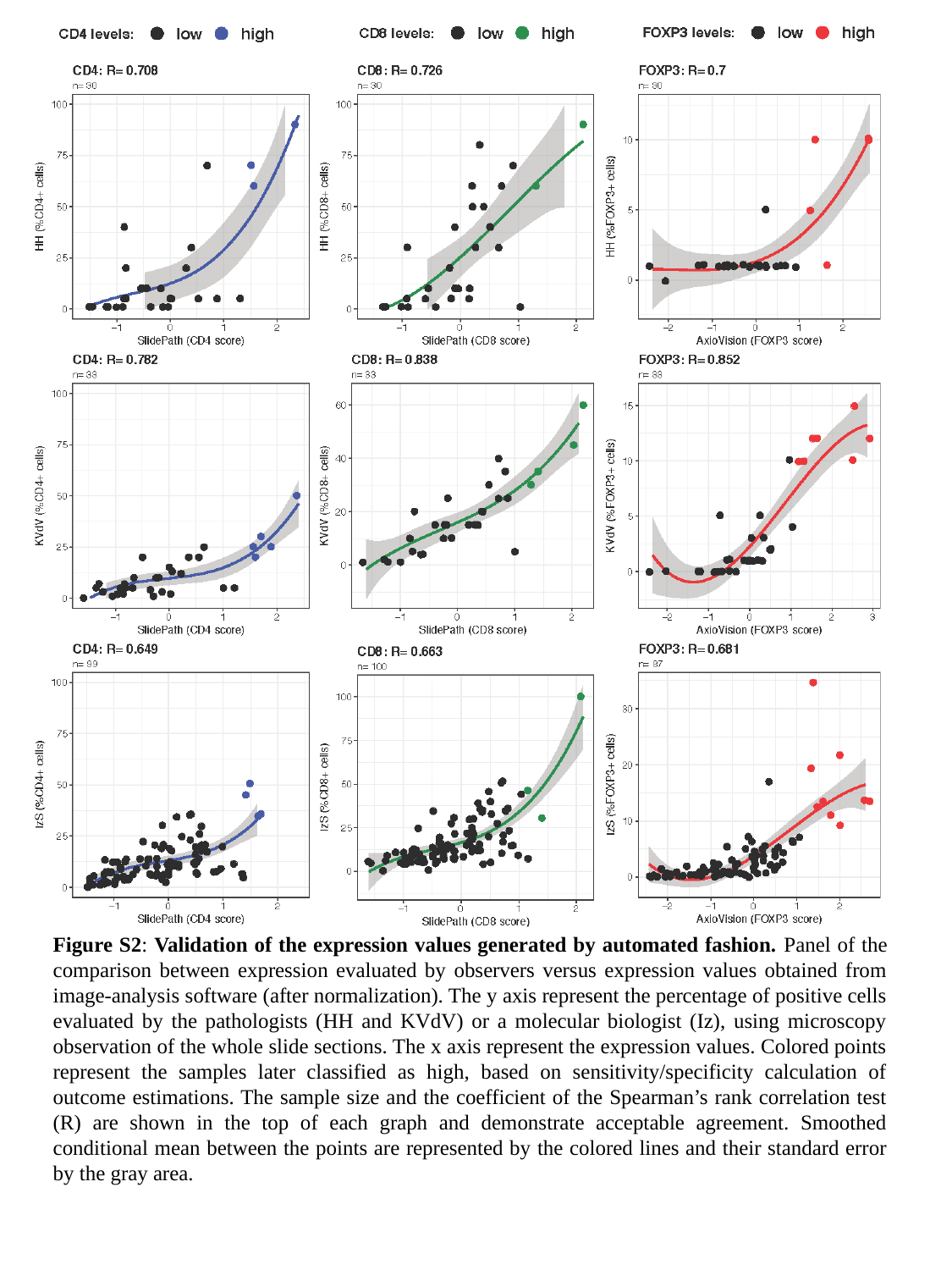

Figure S2: Validation of the expression values generated by automated fashion. Panel of the comparison between expression evaluated by observers versus expression values obtained from image-analysis software (after normalization). The y axis represent the percentage of positive cells evaluated by the pathologists (HH and KVdV) or a molecular biologist (Iz), using microscopy observation of the whole slide sections. The x axis represent the expression values. Colored points represent the samples later classified as high, based on sensitivity/specificity calculation of outcome estimations. The sample size and the coefficient of the Spearman’s rank correlation test (R) are shown in the top of each graph and demonstrate acceptable agreement. Smoothed conditional mean between the points are represented by the colored lines and their standard error by the gray area.

## Slide 4
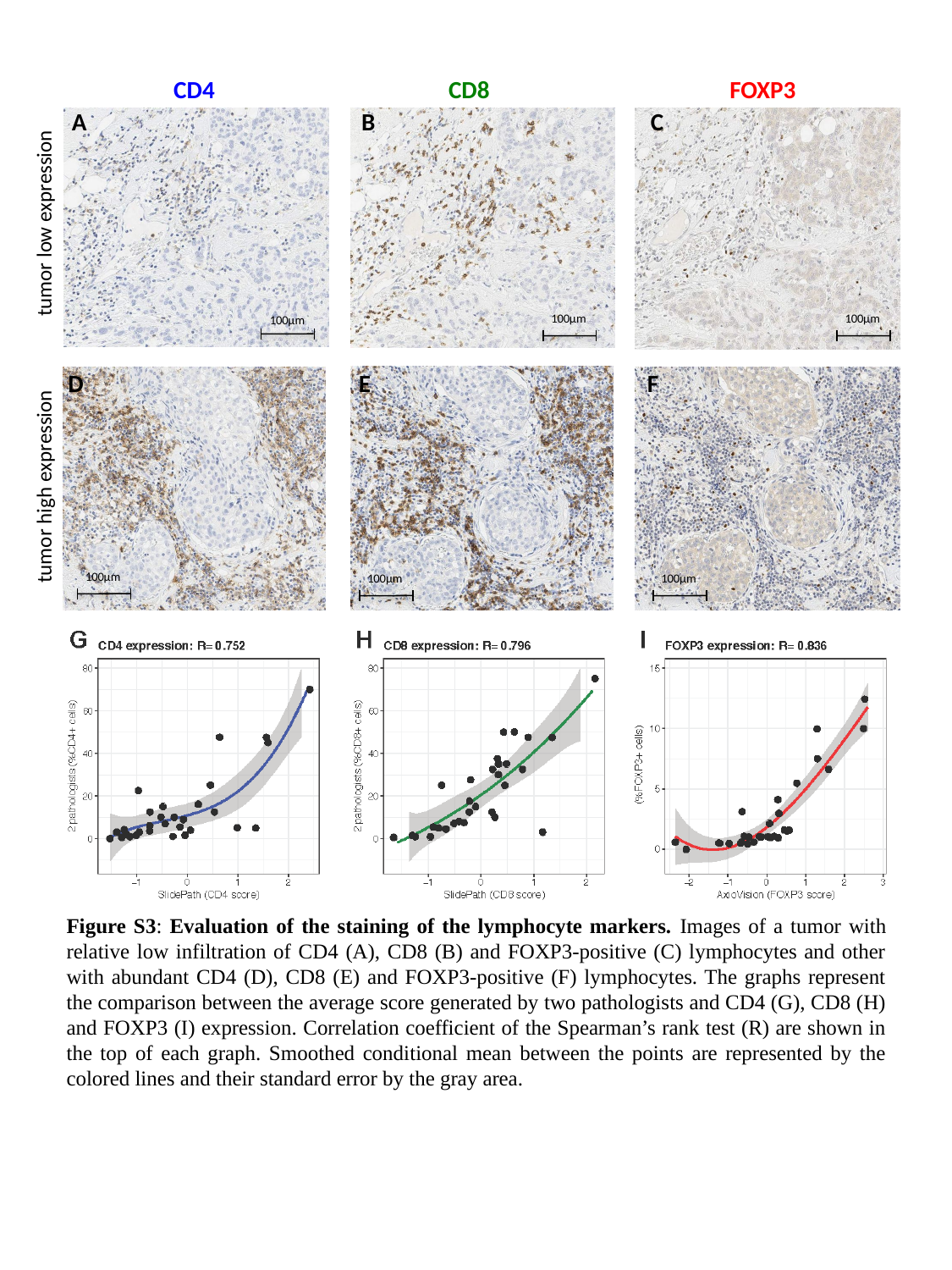

FOXP3
CD4
CD8
A
B
C
tumor low expression
100μm
100μm
100μm
D
E
F
tumor high expression
100μm
100μm
100μm
Figure S3: Evaluation of the staining of the lymphocyte markers. Images of a tumor with relative low infiltration of CD4 (A), CD8 (B) and FOXP3-positive (C) lymphocytes and other with abundant CD4 (D), CD8 (E) and FOXP3-positive (F) lymphocytes. The graphs represent the comparison between the average score generated by two pathologists and CD4 (G), CD8 (H) and FOXP3 (I) expression. Correlation coefficient of the Spearman’s rank test (R) are shown in the top of each graph. Smoothed conditional mean between the points are represented by the colored lines and their standard error by the gray area.

## Slide 5
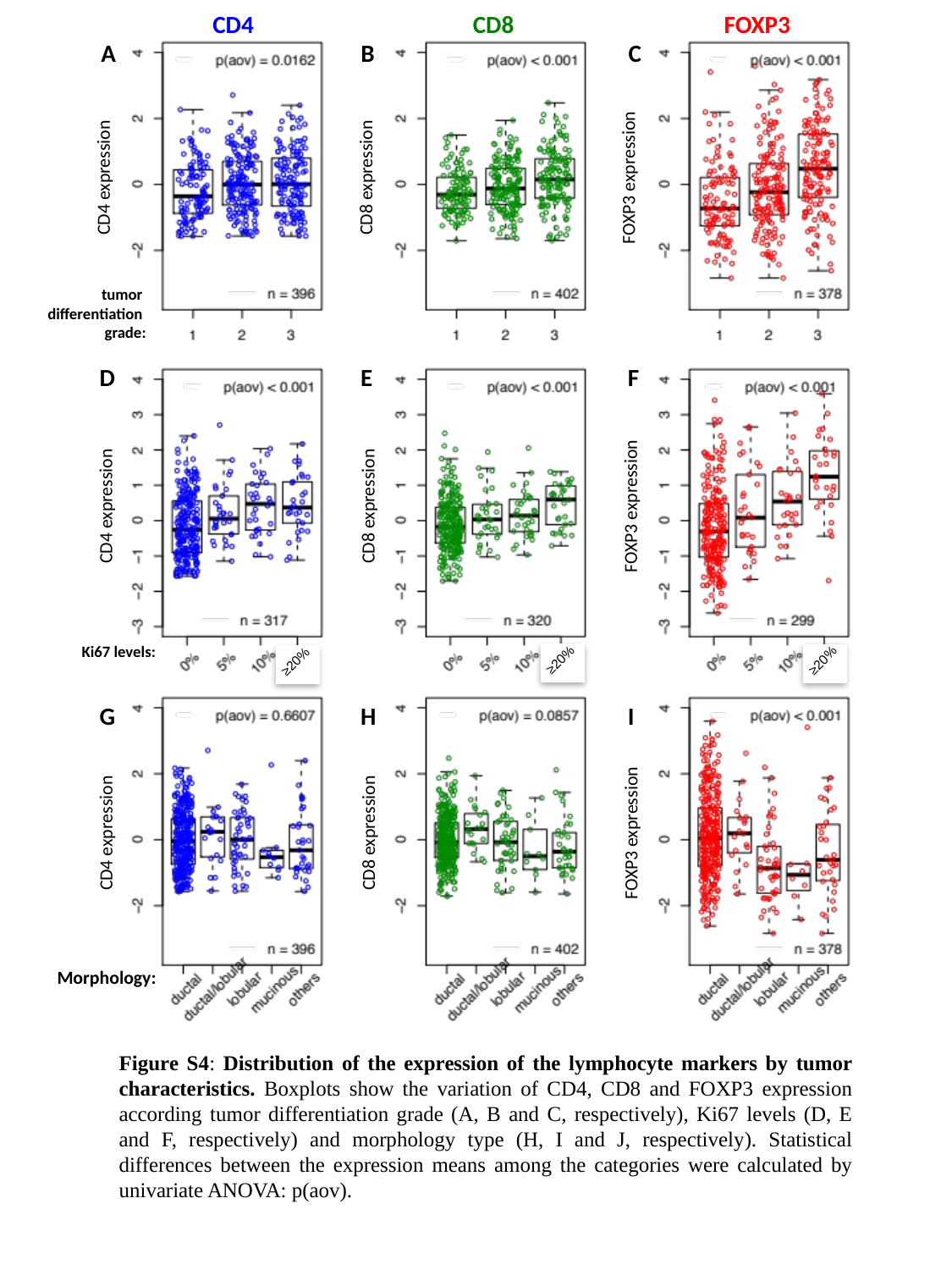

FOXP3
CD4
CD8
A
B
C
CD4 expression
CD8 expression
FOXP3 expression
tumor
differentiation
grade:
D
E
F
CD4 expression
CD8 expression
FOXP3 expression
Ki67 levels:
≥20%
≥20%
≥20%
G
H
I
CD4 expression
CD8 expression
FOXP3 expression
Morphology:
Figure S4: Distribution of the expression of the lymphocyte markers by tumor characteristics. Boxplots show the variation of CD4, CD8 and FOXP3 expression according tumor differentiation grade (A, B and C, respectively), Ki67 levels (D, E and F, respectively) and morphology type (H, I and J, respectively). Statistical differences between the expression means among the categories were calculated by univariate ANOVA: p(aov).

## Slide 6
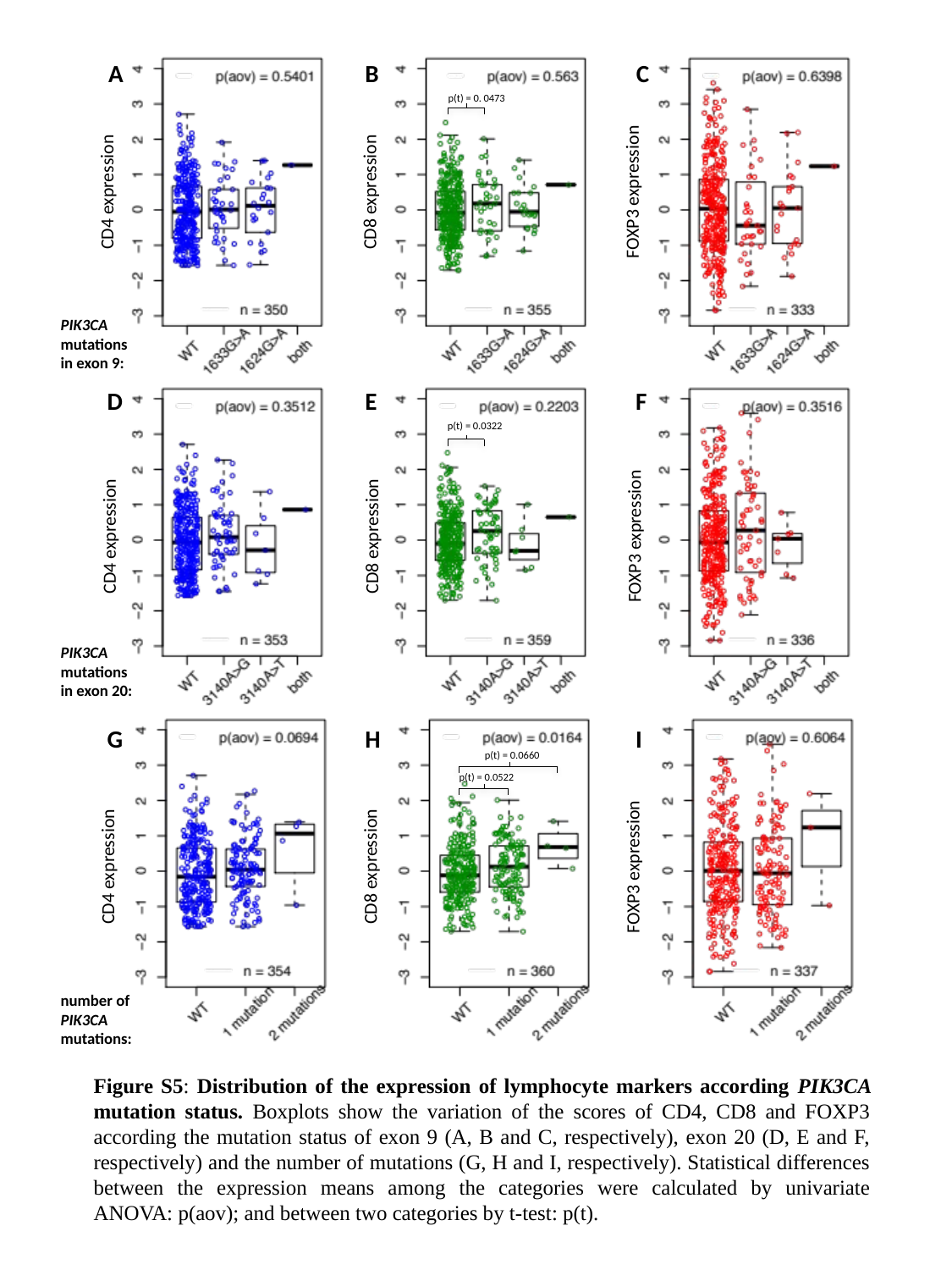

A
B
C
p(t) = 0. 0473
CD4 expression
CD8 expression
FOXP3 expression
PIK3CA mutations in exon 9:
D
E
F
p(t) = 0.0322
CD4 expression
CD8 expression
FOXP3 expression
PIK3CA mutations in exon 20:
G
H
I
p(t) = 0.0660
p(t) = 0.0522
CD4 expression
CD8 expression
FOXP3 expression
number of PIK3CA mutations:
Figure S5: Distribution of the expression of lymphocyte markers according PIK3CA mutation status. Boxplots show the variation of the scores of CD4, CD8 and FOXP3 according the mutation status of exon 9 (A, B and C, respectively), exon 20 (D, E and F, respectively) and the number of mutations (G, H and I, respectively). Statistical differences between the expression means among the categories were calculated by univariate ANOVA: p(aov); and between two categories by t-test: p(t).

## Slide 7
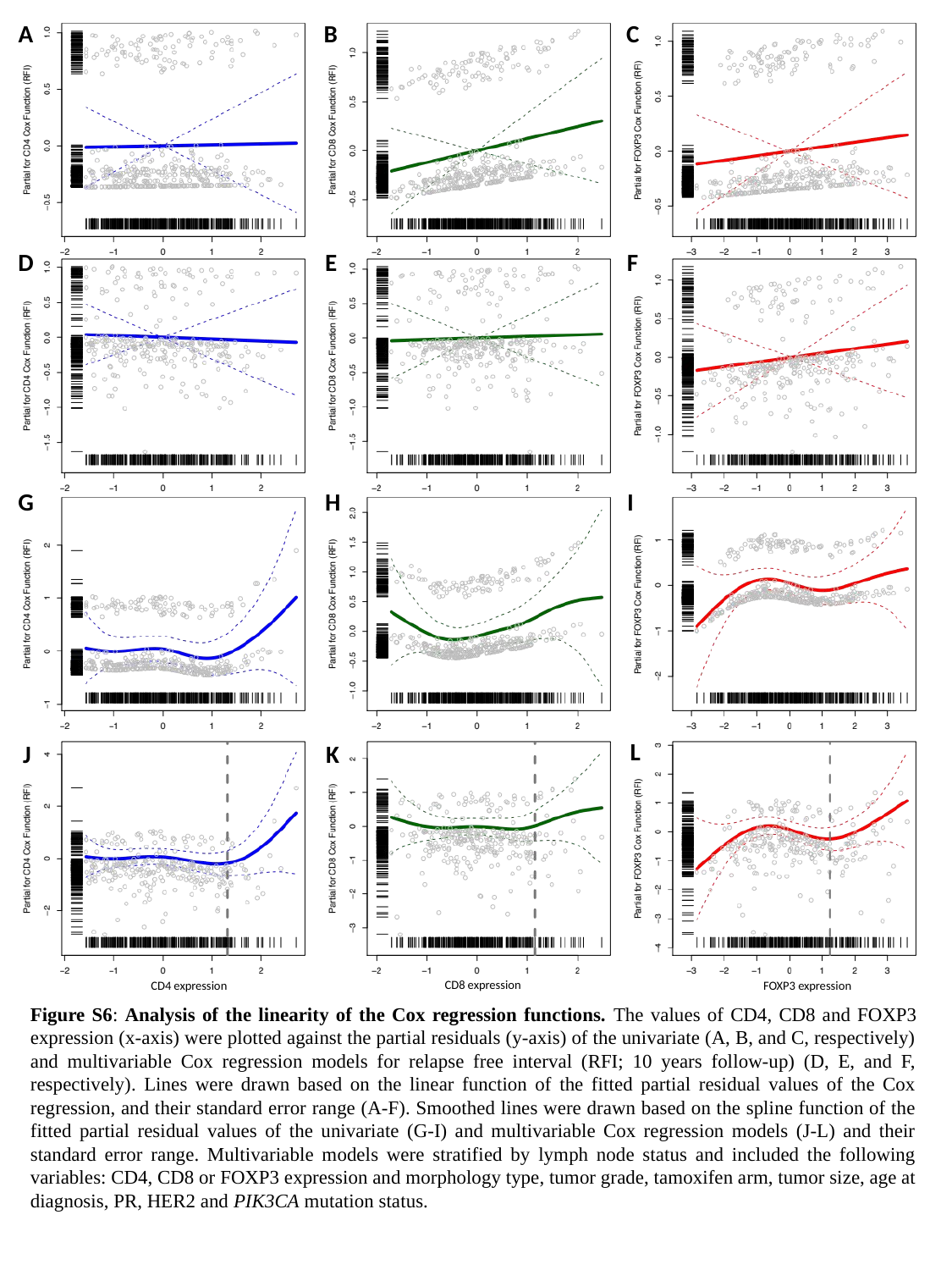

A
B
C
D
E
F
G
H
I
L
J
K
CD8 expression
CD4 expression
FOXP3 expression
Figure S6: Analysis of the linearity of the Cox regression functions. The values of CD4, CD8 and FOXP3 expression (x-axis) were plotted against the partial residuals (y-axis) of the univariate (A, B, and C, respectively) and multivariable Cox regression models for relapse free interval (RFI; 10 years follow-up) (D, E, and F, respectively). Lines were drawn based on the linear function of the fitted partial residual values of the Cox regression, and their standard error range (A-F). Smoothed lines were drawn based on the spline function of the fitted partial residual values of the univariate (G-I) and multivariable Cox regression models (J-L) and their standard error range. Multivariable models were stratified by lymph node status and included the following variables: CD4, CD8 or FOXP3 expression and morphology type, tumor grade, tamoxifen arm, tumor size, age at diagnosis, PR, HER2 and PIK3CA mutation status.

## Slide 8
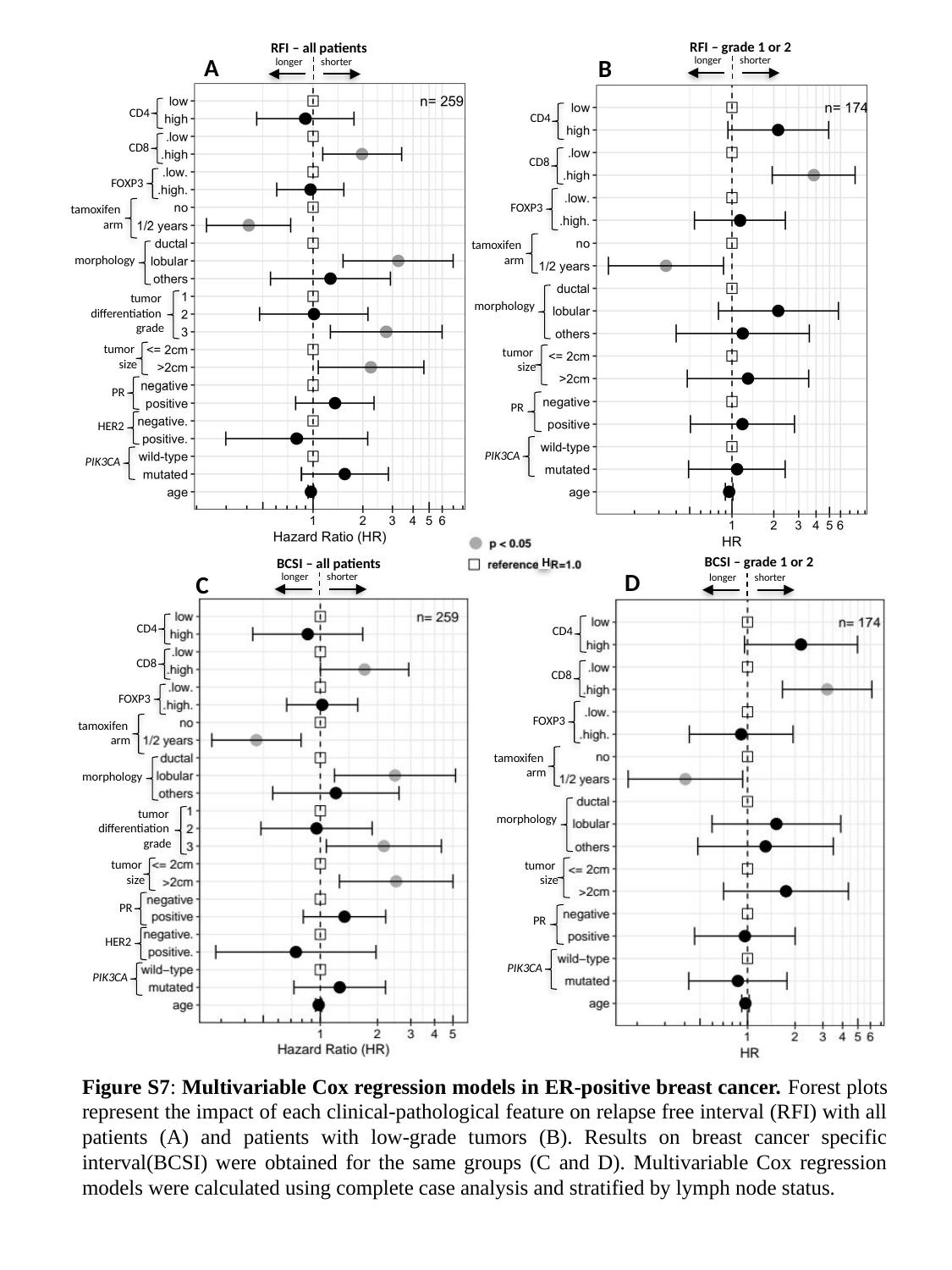

RFI – grade 1 or 2
longer
shorter
RFI – all patients
longer
shorter
A
B
CD4
CD4
CD8
CD8
FOXP3
FOXP3
tamoxifen
arm
tamoxifen
arm
morphology
tumor
differentiation
grade
morphology
tumor
size
tumor
size
PR
PR
HER2
PIK3CA
PIK3CA
H
BCSI – grade 1 or 2
longer
shorter
BCSI – all patients
longer
shorter
D
C
CD4
CD4
CD8
CD8
FOXP3
FOXP3
tamoxifen
arm
tamoxifen
arm
morphology
morphology
tumor
differentiation
grade
tumor
size
tumor
size
PR
PR
HER2
PIK3CA
PIK3CA
Figure S7: Multivariable Cox regression models in ER-positive breast cancer. Forest plots represent the impact of each clinical-pathological feature on relapse free interval (RFI) with all patients (A) and patients with low-grade tumors (B). Results on breast cancer specific interval(BCSI) were obtained for the same groups (C and D). Multivariable Cox regression models were calculated using complete case analysis and stratified by lymph node status.

## Slide 9
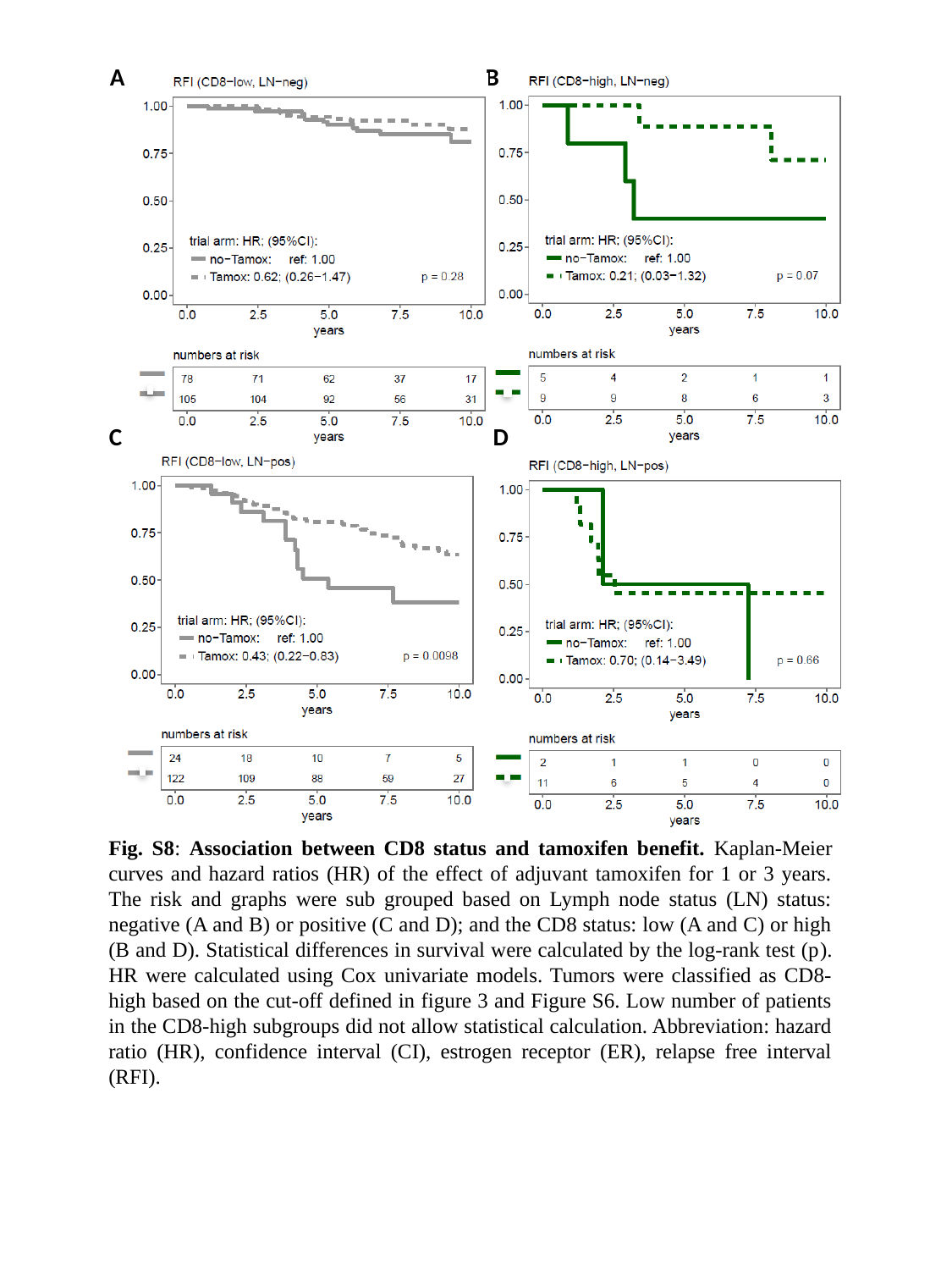

A
B
C
D
Fig. S8: Association between CD8 status and tamoxifen benefit. Kaplan-Meier curves and hazard ratios (HR) of the effect of adjuvant tamoxifen for 1 or 3 years. The risk and graphs were sub grouped based on Lymph node status (LN) status: negative (A and B) or positive (C and D); and the CD8 status: low (A and C) or high (B and D). Statistical differences in survival were calculated by the log-rank test (p). HR were calculated using Cox univariate models. Tumors were classified as CD8-high based on the cut-off defined in figure 3 and Figure S6. Low number of patients in the CD8-high subgroups did not allow statistical calculation. Abbreviation: hazard ratio (HR), confidence interval (CI), estrogen receptor (ER), relapse free interval (RFI).

## Slide 10
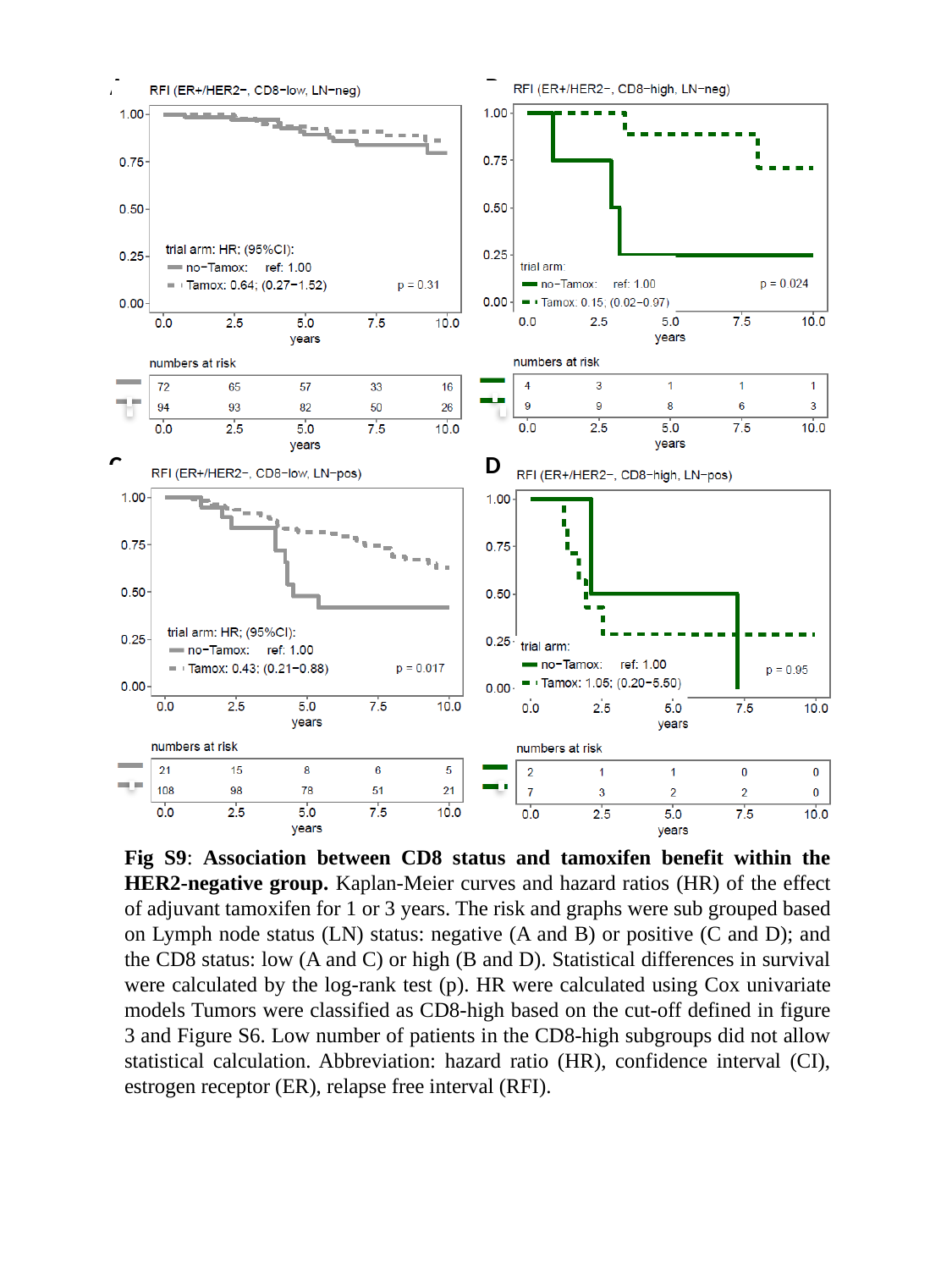

A
B
C
D
Fig S9: Association between CD8 status and tamoxifen benefit within the HER2-negative group. Kaplan-Meier curves and hazard ratios (HR) of the effect of adjuvant tamoxifen for 1 or 3 years. The risk and graphs were sub grouped based on Lymph node status (LN) status: negative (A and B) or positive (C and D); and the CD8 status: low (A and C) or high (B and D). Statistical differences in survival were calculated by the log-rank test (p). HR were calculated using Cox univariate models Tumors were classified as CD8-high based on the cut-off defined in figure 3 and Figure S6. Low number of patients in the CD8-high subgroups did not allow statistical calculation. Abbreviation: hazard ratio (HR), confidence interval (CI), estrogen receptor (ER), relapse free interval (RFI).

## Slide 11
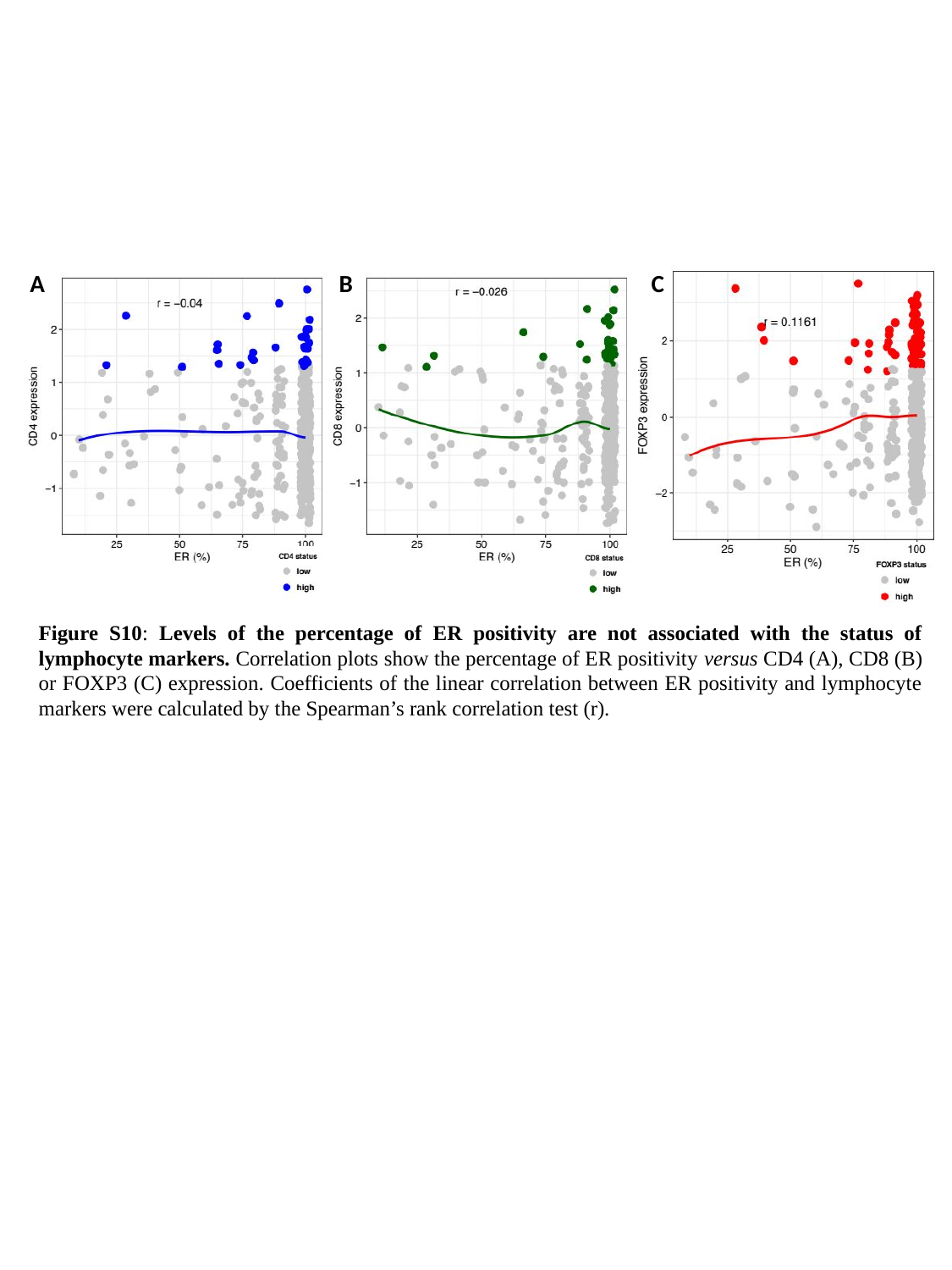

A
B
C
Figure S10: Levels of the percentage of ER positivity are not associated with the status of lymphocyte markers. Correlation plots show the percentage of ER positivity versus CD4 (A), CD8 (B) or FOXP3 (C) expression. Coefficients of the linear correlation between ER positivity and lymphocyte markers were calculated by the Spearman’s rank correlation test (r).
