# Supplementary material for: Cancer-immune interactions in ER-positive breast cancers: PI3K pathway alterations and tumor-infiltrating lymphocytes
Source: Breast Cancer Res. 2019 Aug 7;21:90. doi: 10.1186/s13058-019-1176-2 (PMC6686400; doi:10.1186/s13058-019-1176-2)
Supplement: Supplementary file 2 — Table S1. Staining details. Table S2. Interaction terms. Table S3. PI3K pathway activation and lymphocytic infiltration. Table S4. Comparison between all patients and those with CD4, CD8, or FOXP3 staining. (PPTX 272 kb) [file 13058_2019_1176_MOESM2_ESM.pptx]

## Slide 1
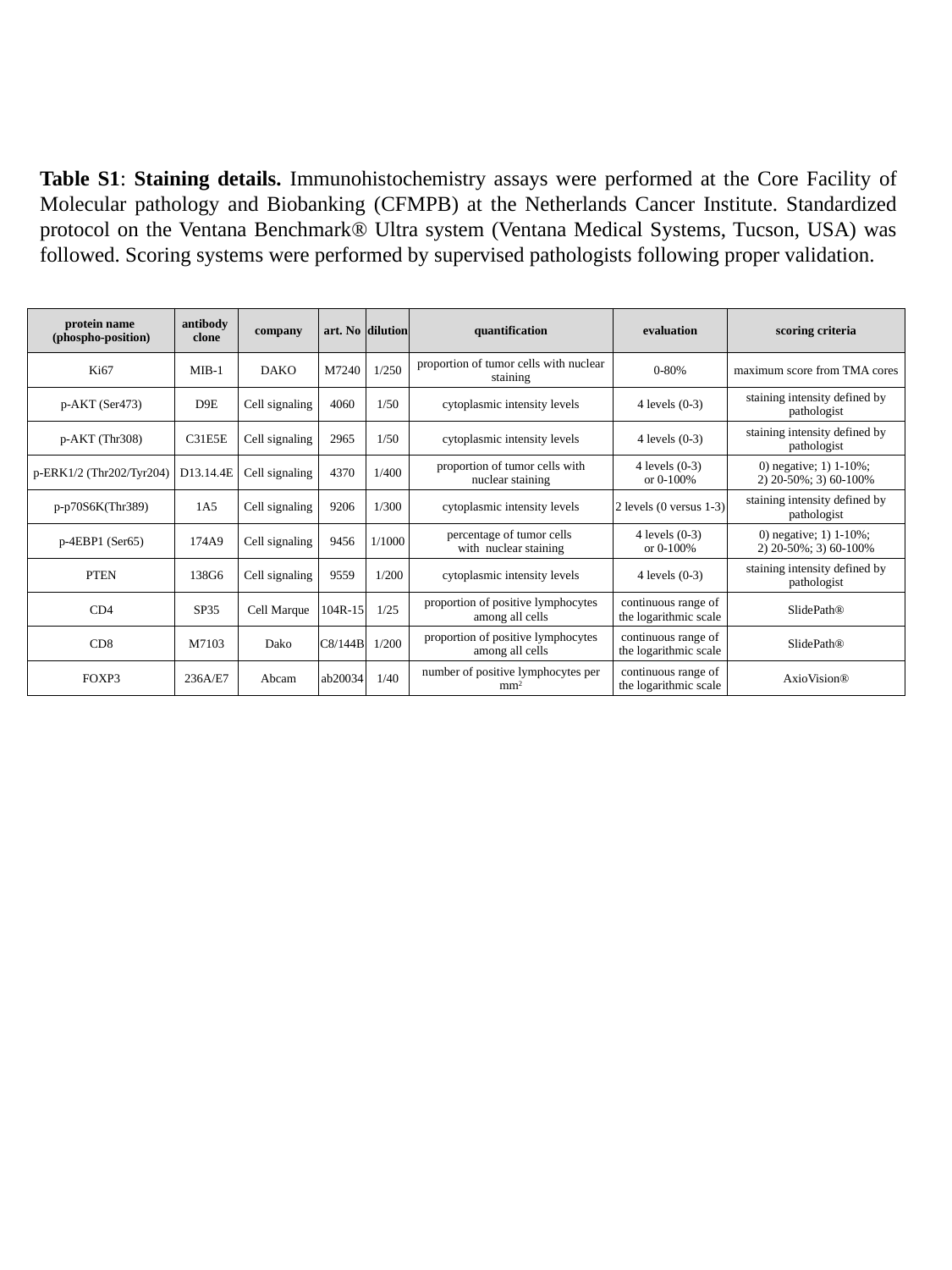

Table S1: Staining details. Immunohistochemistry assays were performed at the Core Facility of Molecular pathology and Biobanking (CFMPB) at the Netherlands Cancer Institute. Standardized protocol on the Ventana Benchmark® Ultra system (Ventana Medical Systems, Tucson, USA) was followed. Scoring systems were performed by supervised pathologists following proper validation.
| protein name (phospho-position) | antibody clone | company | art. No | dilution | quantification | evaluation | scoring criteria |
| --- | --- | --- | --- | --- | --- | --- | --- |
| Ki67 | MIB-1 | DAKO | M7240 | 1/250 | proportion of tumor cells with nuclear staining | 0-80% | maximum score from TMA cores |
| p-AKT (Ser473) | D9E | Cell signaling | 4060 | 1/50 | cytoplasmic intensity levels | 4 levels (0-3) | staining intensity defined by pathologist |
| p-AKT (Thr308) | C31E5E | Cell signaling | 2965 | 1/50 | cytoplasmic intensity levels | 4 levels (0-3) | staining intensity defined by pathologist |
| p-ERK1/2 (Thr202/Tyr204) | D13.14.4E | Cell signaling | 4370 | 1/400 | proportion of tumor cells with nuclear staining | 4 levels (0-3) or 0-100% | 0) negative; 1) 1-10%; 2) 20-50%; 3) 60-100% |
| p-p70S6K(Thr389) | 1A5 | Cell signaling | 9206 | 1/300 | cytoplasmic intensity levels | 2 levels (0 versus 1-3) | staining intensity defined by pathologist |
| p-4EBP1 (Ser65) | 174A9 | Cell signaling | 9456 | 1/1000 | percentage of tumor cells with nuclear staining | 4 levels (0-3) or 0-100% | 0) negative; 1) 1-10%; 2) 20-50%; 3) 60-100% |
| PTEN | 138G6 | Cell signaling | 9559 | 1/200 | cytoplasmic intensity levels | 4 levels (0-3) | staining intensity defined by pathologist |
| CD4 | SP35 | Cell Marque | 104R-15 | 1/25 | proportion of positive lymphocytes among all cells | continuous range of the logarithmic scale | SlidePath® |
| CD8 | M7103 | Dako | C8/144B | 1/200 | proportion of positive lymphocytes among all cells | continuous range of the logarithmic scale | SlidePath® |
| FOXP3 | 236A/E7 | Abcam | ab20034 | 1/40 | number of positive lymphocytes per mm2 | continuous range of the logarithmic scale | AxioVision® |

## Slide 2
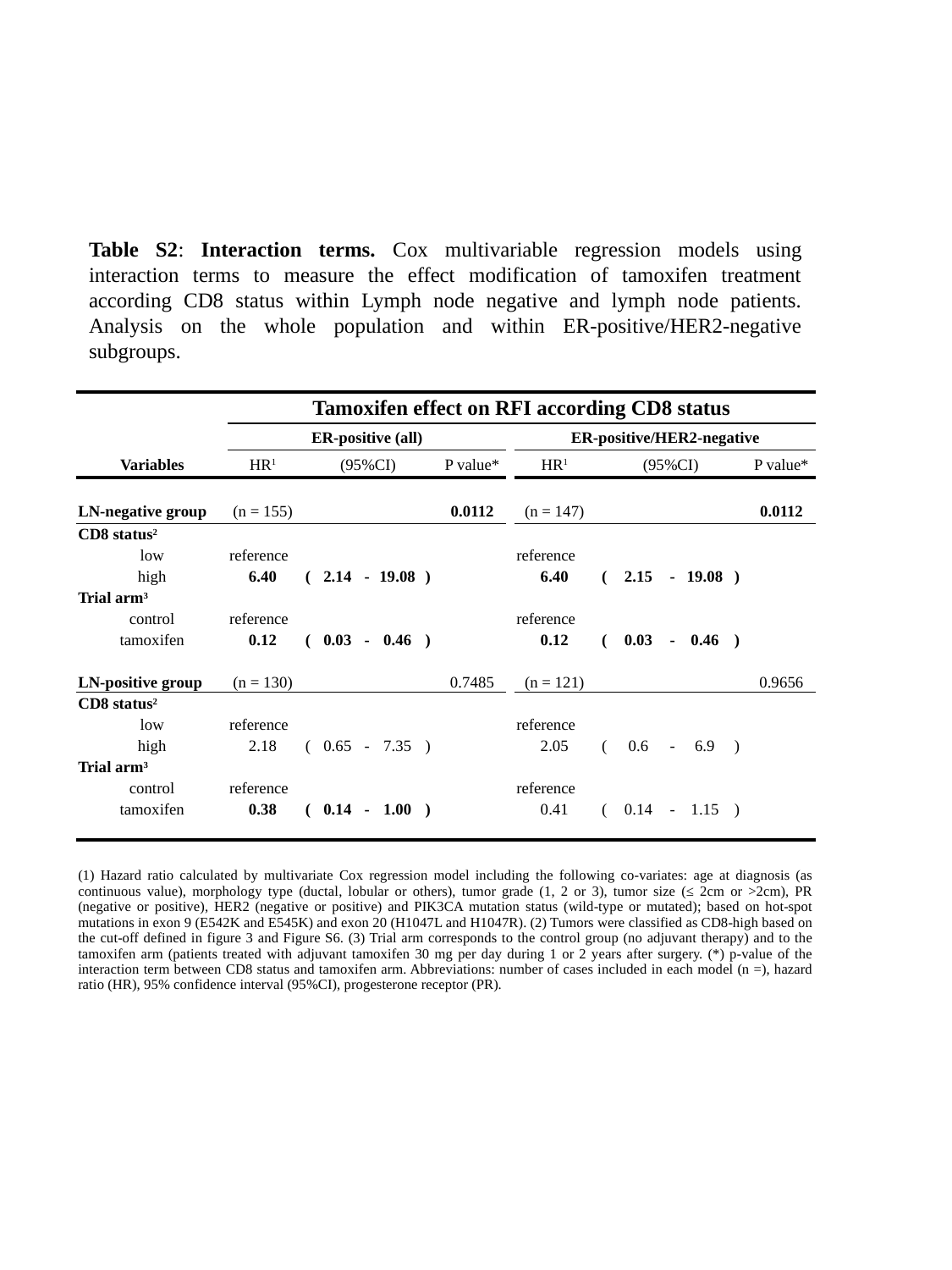

Table S2: Interaction terms. Cox multivariable regression models using interaction terms to measure the effect modification of tamoxifen treatment according CD8 status within Lymph node negative and lymph node patients. Analysis on the whole population and within ER-positive/HER2-negative subgroups.
| | | | | | | | | | | | | | | | |
| --- | --- | --- | --- | --- | --- | --- | --- | --- | --- | --- | --- | --- | --- | --- | --- |
| | Tamoxifen effect on RFI according CD8 status | | | | | | | | | | | | | | |
| | ER-positive (all) | | | | | | | | ER-positive/HER2-negative | | | | | | |
| Variables | HR1 | | (95%CI) | | | | P value\* | | HR1 | | (95%CI) | | | | P value\* |
| | | | | | | | | | | | | | | | |
| LN-negative group | (n = 155) | | | | | | 0.0112 | | (n = 147) | | | | | | 0.0112 |
| CD8 status2 | | | | | | | | | | | | | | | |
| low | reference | | | | | | | | reference | | | | | | |
| high | 6.40 | ( | 2.14 | - | 19.08 | ) | | | 6.40 | ( | 2.15 | - | 19.08 | ) | |
| Trial arm3 | | | | | | | | | | | | | | | |
| control | reference | | | | | | | | reference | | | | | | |
| tamoxifen | 0.12 | ( | 0.03 | - | 0.46 | ) | | | 0.12 | ( | 0.03 | - | 0.46 | ) | |
| | | | | | | | | | | | | | | | |
| LN-positive group | (n = 130) | | | | | | 0.7485 | | (n = 121) | | | | | | 0.9656 |
| CD8 status2 | | | | | | | | | | | | | | | |
| low | reference | | | | | | | | reference | | | | | | |
| high | 2.18 | ( | 0.65 | - | 7.35 | ) | | | 2.05 | ( | 0.6 | - | 6.9 | ) | |
| Trial arm3 | | | | | | | | | | | | | | | |
| control | reference | | | | | | | | reference | | | | | | |
| tamoxifen | 0.38 | ( | 0.14 | - | 1.00 | ) | | | 0.41 | ( | 0.14 | - | 1.15 | ) | |
| | | | | | | | | | | | | | | | |
| | | | | | | | | | | | | | | | |
| (1) Hazard ratio calculated by multivariate Cox regression model including the following co-variates: age at diagnosis (as continuous value), morphology type (ductal, lobular or others), tumor grade (1, 2 or 3), tumor size (≤ 2cm or >2cm), PR (negative or positive), HER2 (negative or positive) and PIK3CA mutation status (wild-type or mutated); based on hot-spot mutations in exon 9 (E542K and E545K) and exon 20 (H1047L and H1047R). (2) Tumors were classified as CD8-high based on the cut-off defined in figure 3 and Figure S6. (3) Trial arm corresponds to the control group (no adjuvant therapy) and to the tamoxifen arm (patients treated with adjuvant tamoxifen 30 mg per day during 1 or 2 years after surgery. (\*) p-value of the interaction term between CD8 status and tamoxifen arm. Abbreviations: number of cases included in each model (n =), hazard ratio (HR), 95% confidence interval (95%CI), progesterone receptor (PR). | | | | | | | | | | | | | | | |

## Slide 3
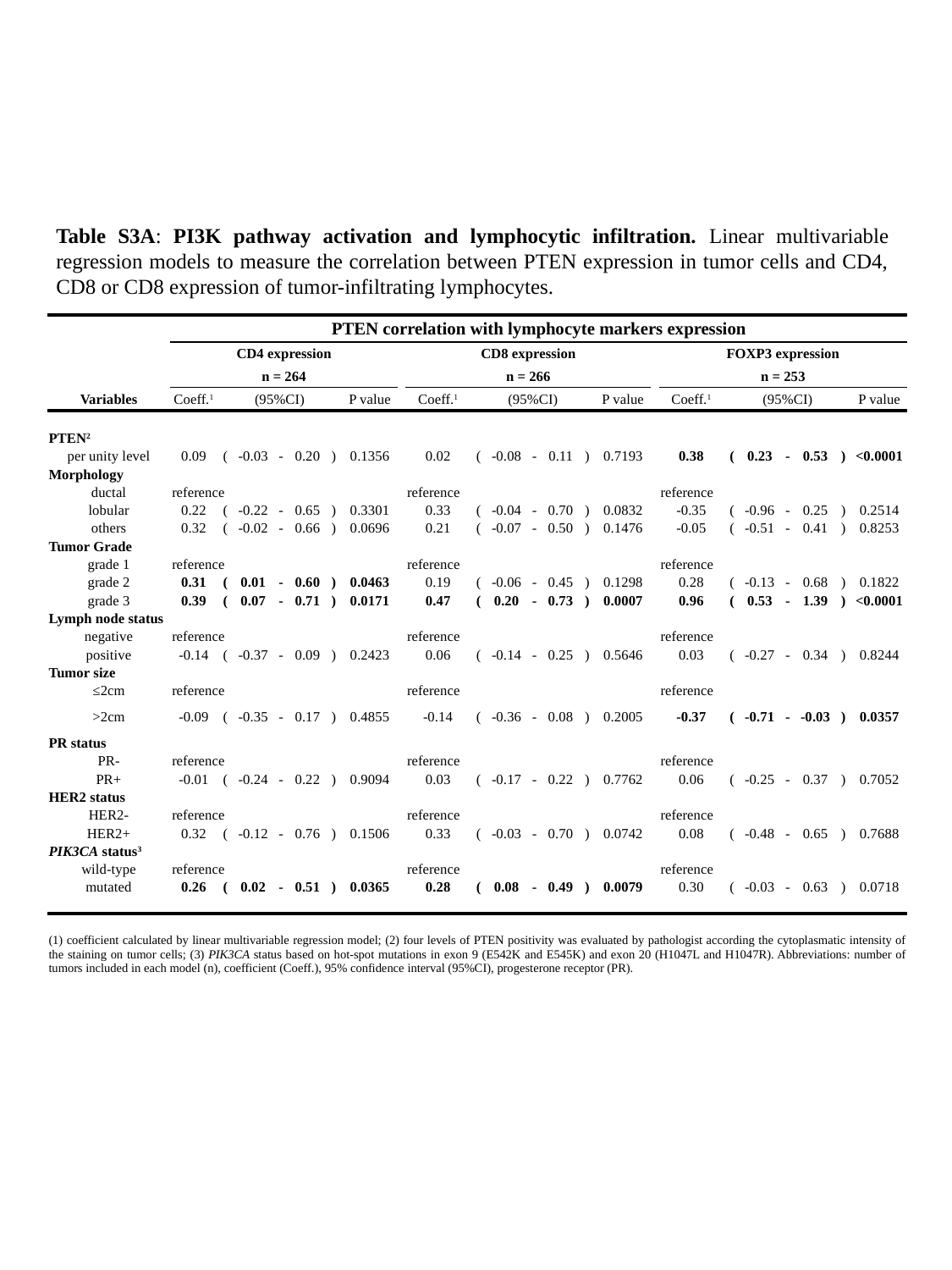

Table S3A: PI3K pathway activation and lymphocytic infiltration. Linear multivariable regression models to measure the correlation between PTEN expression in tumor cells and CD4, CD8 or CD8 expression of tumor-infiltrating lymphocytes.
| | PTEN correlation with lymphocyte markers expression | | | | | | | | | | | | | | | | | | | | | | |
| --- | --- | --- | --- | --- | --- | --- | --- | --- | --- | --- | --- | --- | --- | --- | --- | --- | --- | --- | --- | --- | --- | --- | --- |
| | CD4 expression | | | | | | | | CD8 expression | | | | | | | | FOXP3 expression | | | | | | |
| | n = 264 | | | | | | | | n = 266 | | | | | | | | n = 253 | | | | | | |
| Variables | Coeff.1 | | (95%CI) | | | | P value | | Coeff.1 | | (95%CI) | | | | P value | | Coeff.1 | | (95%CI) | | | | P value |
| | | | | | | | | | | | | | | | | | | | | | | | |
| PTEN2 | | | | | | | | | | | | | | | | | | | | | | | |
| per unity level | 0.09 | ( | -0.03 | - | 0.20 | ) | 0.1356 | | 0.02 | ( | -0.08 | - | 0.11 | ) | 0.7193 | | 0.38 | ( | 0.23 | - | 0.53 | ) | <0.0001 |
| Morphology | | | | | | | | | | | | | | | | | | | | | | | |
| ductal | reference | | | | | | | | reference | | | | | | | | reference | | | | | | |
| lobular | 0.22 | ( | -0.22 | - | 0.65 | ) | 0.3301 | | 0.33 | ( | -0.04 | - | 0.70 | ) | 0.0832 | | -0.35 | ( | -0.96 | - | 0.25 | ) | 0.2514 |
| others | 0.32 | ( | -0.02 | - | 0.66 | ) | 0.0696 | | 0.21 | ( | -0.07 | - | 0.50 | ) | 0.1476 | | -0.05 | ( | -0.51 | - | 0.41 | ) | 0.8253 |
| Tumor Grade | | | | | | | | | | | | | | | | | | | | | | | |
| grade 1 | reference | | | | | | | | reference | | | | | | | | reference | | | | | | |
| grade 2 | 0.31 | ( | 0.01 | - | 0.60 | ) | 0.0463 | | 0.19 | ( | -0.06 | - | 0.45 | ) | 0.1298 | | 0.28 | ( | -0.13 | - | 0.68 | ) | 0.1822 |
| grade 3 | 0.39 | ( | 0.07 | - | 0.71 | ) | 0.0171 | | 0.47 | ( | 0.20 | - | 0.73 | ) | 0.0007 | | 0.96 | ( | 0.53 | - | 1.39 | ) | <0.0001 |
| Lymph node status | | | | | | | | | | | | | | | | | | | | | | | |
| negative | reference | | | | | | | | reference | | | | | | | | reference | | | | | | |
| positive | -0.14 | ( | -0.37 | - | 0.09 | ) | 0.2423 | | 0.06 | ( | -0.14 | - | 0.25 | ) | 0.5646 | | 0.03 | ( | -0.27 | - | 0.34 | ) | 0.8244 |
| Tumor size | | | | | | | | | | | | | | | | | | | | | | | |
| ≤2cm | reference | | | | | | | | reference | | | | | | | | reference | | | | | | |
| >2cm | -0.09 | ( | -0.35 | - | 0.17 | ) | 0.4855 | | -0.14 | ( | -0.36 | - | 0.08 | ) | 0.2005 | | -0.37 | ( | -0.71 | - | -0.03 | ) | 0.0357 |
| PR status | | | | | | | | | | | | | | | | | | | | | | | |
| PR- | reference | | | | | | | | reference | | | | | | | | reference | | | | | | |
| PR+ | -0.01 | ( | -0.24 | - | 0.22 | ) | 0.9094 | | 0.03 | ( | -0.17 | - | 0.22 | ) | 0.7762 | | 0.06 | ( | -0.25 | - | 0.37 | ) | 0.7052 |
| HER2 status | | | | | | | | | | | | | | | | | | | | | | | |
| HER2- | reference | | | | | | | | reference | | | | | | | | reference | | | | | | |
| HER2+ | 0.32 | ( | -0.12 | - | 0.76 | ) | 0.1506 | | 0.33 | ( | -0.03 | - | 0.70 | ) | 0.0742 | | 0.08 | ( | -0.48 | - | 0.65 | ) | 0.7688 |
| PIK3CA status3 | | | | | | | | | | | | | | | | | | | | | | | |
| wild-type | reference | | | | | | | | reference | | | | | | | | reference | | | | | | |
| mutated | 0.26 | ( | 0.02 | - | 0.51 | ) | 0.0365 | | 0.28 | ( | 0.08 | - | 0.49 | ) | 0.0079 | | 0.30 | ( | -0.03 | - | 0.63 | ) | 0.0718 |
| | | | | | | | | | | | | | | | | | | | | | | | |
| | | | | | | | | | | | | | | | | | | | | | | | |
| (1) coefficient calculated by linear multivariable regression model; (2) four levels of PTEN positivity was evaluated by pathologist according the cytoplasmatic intensity of the staining on tumor cells; (3) PIK3CA status based on hot-spot mutations in exon 9 (E542K and E545K) and exon 20 (H1047L and H1047R). Abbreviations: number of tumors included in each model (n), coefficient (Coeff.), 95% confidence interval (95%CI), progesterone receptor (PR). | | | | | | | | | | | | | | | | | | | | | | | |

## Slide 4
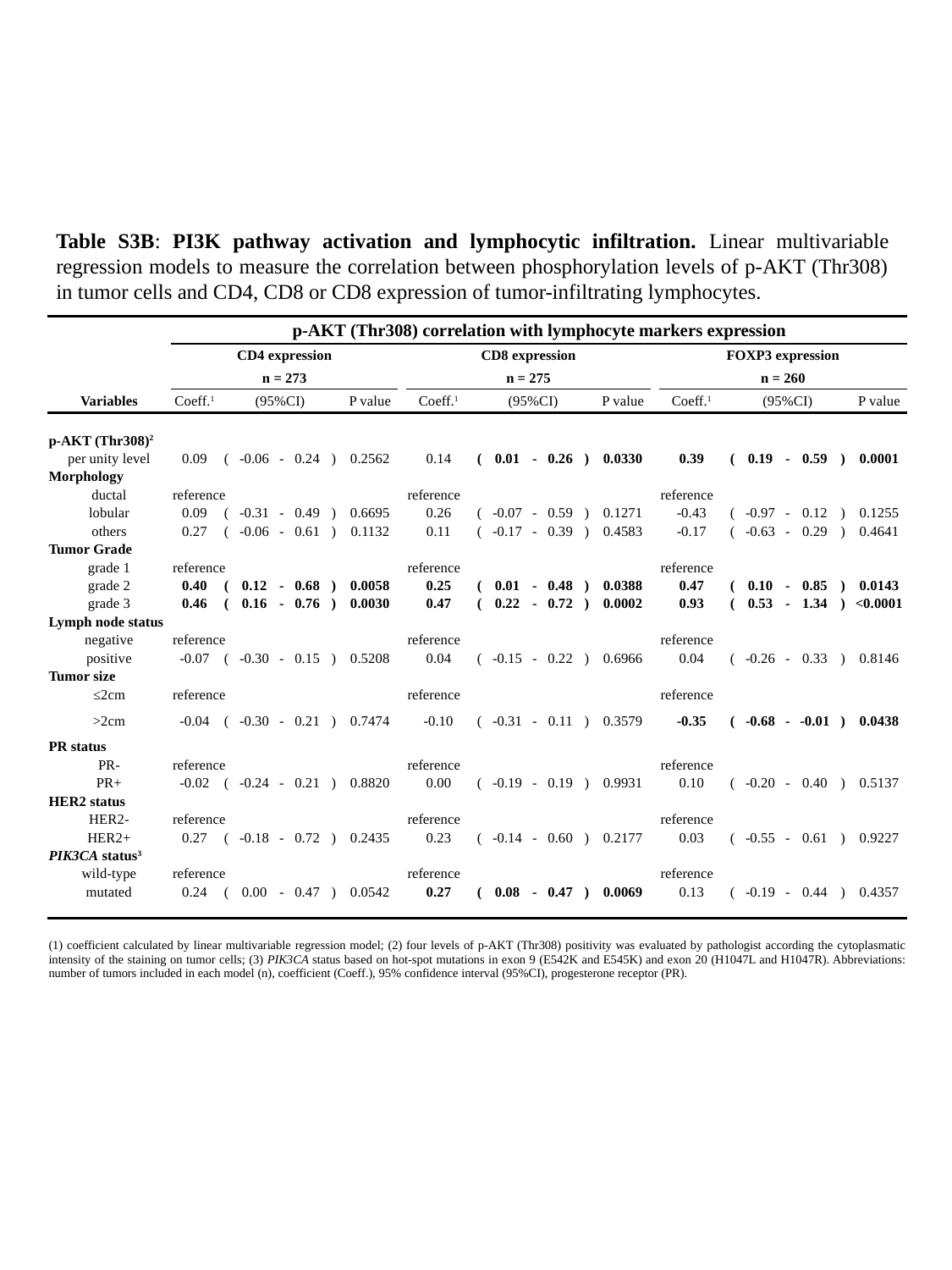

Table S3B: PI3K pathway activation and lymphocytic infiltration. Linear multivariable regression models to measure the correlation between phosphorylation levels of p-AKT (Thr308) in tumor cells and CD4, CD8 or CD8 expression of tumor-infiltrating lymphocytes.
| | p-AKT (Thr308) correlation with lymphocyte markers expression | | | | | | | | | | | | | | | | | | | | | | |
| --- | --- | --- | --- | --- | --- | --- | --- | --- | --- | --- | --- | --- | --- | --- | --- | --- | --- | --- | --- | --- | --- | --- | --- |
| | CD4 expression | | | | | | | | CD8 expression | | | | | | | | FOXP3 expression | | | | | | |
| | n = 273 | | | | | | | | n = 275 | | | | | | | | n = 260 | | | | | | |
| Variables | Coeff.1 | | (95%CI) | | | | P value | | Coeff.1 | | (95%CI) | | | | P value | | Coeff.1 | | (95%CI) | | | | P value |
| | | | | | | | | | | | | | | | | | | | | | | | |
| p-AKT (Thr308)2 | | | | | | | | | | | | | | | | | | | | | | | |
| per unity level | 0.09 | ( | -0.06 | - | 0.24 | ) | 0.2562 | | 0.14 | ( | 0.01 | - | 0.26 | ) | 0.0330 | | 0.39 | ( | 0.19 | - | 0.59 | ) | 0.0001 |
| Morphology | | | | | | | | | | | | | | | | | | | | | | | |
| ductal | reference | | | | | | | | reference | | | | | | | | reference | | | | | | |
| lobular | 0.09 | ( | -0.31 | - | 0.49 | ) | 0.6695 | | 0.26 | ( | -0.07 | - | 0.59 | ) | 0.1271 | | -0.43 | ( | -0.97 | - | 0.12 | ) | 0.1255 |
| others | 0.27 | ( | -0.06 | - | 0.61 | ) | 0.1132 | | 0.11 | ( | -0.17 | - | 0.39 | ) | 0.4583 | | -0.17 | ( | -0.63 | - | 0.29 | ) | 0.4641 |
| Tumor Grade | | | | | | | | | | | | | | | | | | | | | | | |
| grade 1 | reference | | | | | | | | reference | | | | | | | | reference | | | | | | |
| grade 2 | 0.40 | ( | 0.12 | - | 0.68 | ) | 0.0058 | | 0.25 | ( | 0.01 | - | 0.48 | ) | 0.0388 | | 0.47 | ( | 0.10 | - | 0.85 | ) | 0.0143 |
| grade 3 | 0.46 | ( | 0.16 | - | 0.76 | ) | 0.0030 | | 0.47 | ( | 0.22 | - | 0.72 | ) | 0.0002 | | 0.93 | ( | 0.53 | - | 1.34 | ) | <0.0001 |
| Lymph node status | | | | | | | | | | | | | | | | | | | | | | | |
| negative | reference | | | | | | | | reference | | | | | | | | reference | | | | | | |
| positive | -0.07 | ( | -0.30 | - | 0.15 | ) | 0.5208 | | 0.04 | ( | -0.15 | - | 0.22 | ) | 0.6966 | | 0.04 | ( | -0.26 | - | 0.33 | ) | 0.8146 |
| Tumor size | | | | | | | | | | | | | | | | | | | | | | | |
| ≤2cm | reference | | | | | | | | reference | | | | | | | | reference | | | | | | |
| >2cm | -0.04 | ( | -0.30 | - | 0.21 | ) | 0.7474 | | -0.10 | ( | -0.31 | - | 0.11 | ) | 0.3579 | | -0.35 | ( | -0.68 | - | -0.01 | ) | 0.0438 |
| PR status | | | | | | | | | | | | | | | | | | | | | | | |
| PR- | reference | | | | | | | | reference | | | | | | | | reference | | | | | | |
| PR+ | -0.02 | ( | -0.24 | - | 0.21 | ) | 0.8820 | | 0.00 | ( | -0.19 | - | 0.19 | ) | 0.9931 | | 0.10 | ( | -0.20 | - | 0.40 | ) | 0.5137 |
| HER2 status | | | | | | | | | | | | | | | | | | | | | | | |
| HER2- | reference | | | | | | | | reference | | | | | | | | reference | | | | | | |
| HER2+ | 0.27 | ( | -0.18 | - | 0.72 | ) | 0.2435 | | 0.23 | ( | -0.14 | - | 0.60 | ) | 0.2177 | | 0.03 | ( | -0.55 | - | 0.61 | ) | 0.9227 |
| PIK3CA status3 | | | | | | | | | | | | | | | | | | | | | | | |
| wild-type | reference | | | | | | | | reference | | | | | | | | reference | | | | | | |
| mutated | 0.24 | ( | 0.00 | - | 0.47 | ) | 0.0542 | | 0.27 | ( | 0.08 | - | 0.47 | ) | 0.0069 | | 0.13 | ( | -0.19 | - | 0.44 | ) | 0.4357 |
| | | | | | | | | | | | | | | | | | | | | | | | |
| | | | | | | | | | | | | | | | | | | | | | | | |
| (1) coefficient calculated by linear multivariable regression model; (2) four levels of p-AKT (Thr308) positivity was evaluated by pathologist according the cytoplasmatic intensity of the staining on tumor cells; (3) PIK3CA status based on hot-spot mutations in exon 9 (E542K and E545K) and exon 20 (H1047L and H1047R). Abbreviations: number of tumors included in each model (n), coefficient (Coeff.), 95% confidence interval (95%CI), progesterone receptor (PR). | | | | | | | | | | | | | | | | | | | | | | | |

## Slide 5
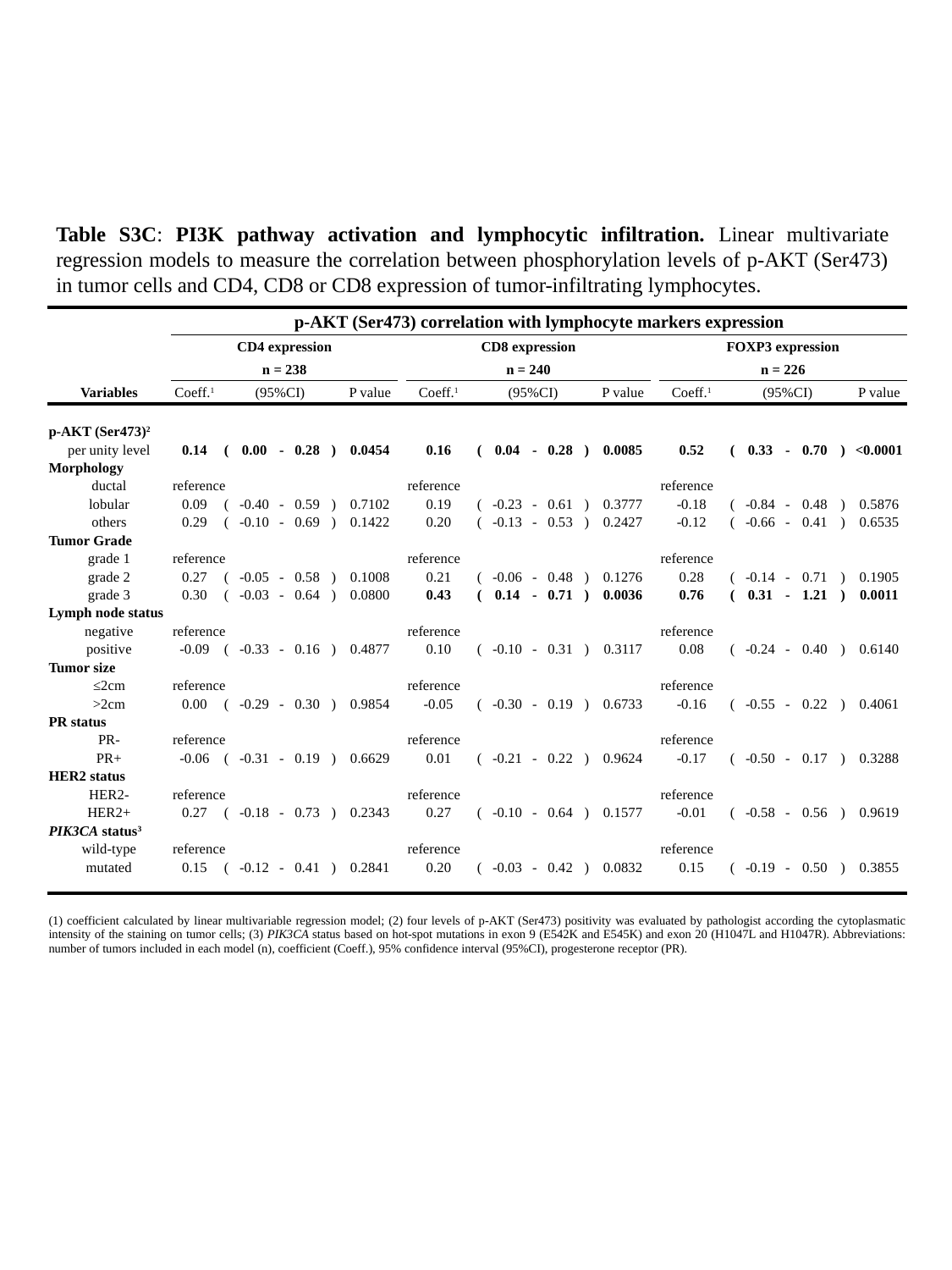

Table S3C: PI3K pathway activation and lymphocytic infiltration. Linear multivariate regression models to measure the correlation between phosphorylation levels of p-AKT (Ser473) in tumor cells and CD4, CD8 or CD8 expression of tumor-infiltrating lymphocytes.
| | p-AKT (Ser473) correlation with lymphocyte markers expression | | | | | | | | | | | | | | | | | | | | | | |
| --- | --- | --- | --- | --- | --- | --- | --- | --- | --- | --- | --- | --- | --- | --- | --- | --- | --- | --- | --- | --- | --- | --- | --- |
| | CD4 expression | | | | | | | | CD8 expression | | | | | | | | FOXP3 expression | | | | | | |
| | n = 238 | | | | | | | | n = 240 | | | | | | | | n = 226 | | | | | | |
| Variables | Coeff.1 | | (95%CI) | | | | P value | | Coeff.1 | | (95%CI) | | | | P value | | Coeff.1 | | (95%CI) | | | | P value |
| | | | | | | | | | | | | | | | | | | | | | | | |
| p-AKT (Ser473)2 | | | | | | | | | | | | | | | | | | | | | | | |
| per unity level | 0.14 | ( | 0.00 | - | 0.28 | ) | 0.0454 | | 0.16 | ( | 0.04 | - | 0.28 | ) | 0.0085 | | 0.52 | ( | 0.33 | - | 0.70 | ) | <0.0001 |
| Morphology | | | | | | | | | | | | | | | | | | | | | | | |
| ductal | reference | | | | | | | | reference | | | | | | | | reference | | | | | | |
| lobular | 0.09 | ( | -0.40 | - | 0.59 | ) | 0.7102 | | 0.19 | ( | -0.23 | - | 0.61 | ) | 0.3777 | | -0.18 | ( | -0.84 | - | 0.48 | ) | 0.5876 |
| others | 0.29 | ( | -0.10 | - | 0.69 | ) | 0.1422 | | 0.20 | ( | -0.13 | - | 0.53 | ) | 0.2427 | | -0.12 | ( | -0.66 | - | 0.41 | ) | 0.6535 |
| Tumor Grade | | | | | | | | | | | | | | | | | | | | | | | |
| grade 1 | reference | | | | | | | | reference | | | | | | | | reference | | | | | | |
| grade 2 | 0.27 | ( | -0.05 | - | 0.58 | ) | 0.1008 | | 0.21 | ( | -0.06 | - | 0.48 | ) | 0.1276 | | 0.28 | ( | -0.14 | - | 0.71 | ) | 0.1905 |
| grade 3 | 0.30 | ( | -0.03 | - | 0.64 | ) | 0.0800 | | 0.43 | ( | 0.14 | - | 0.71 | ) | 0.0036 | | 0.76 | ( | 0.31 | - | 1.21 | ) | 0.0011 |
| Lymph node status | | | | | | | | | | | | | | | | | | | | | | | |
| negative | reference | | | | | | | | reference | | | | | | | | reference | | | | | | |
| positive | -0.09 | ( | -0.33 | - | 0.16 | ) | 0.4877 | | 0.10 | ( | -0.10 | - | 0.31 | ) | 0.3117 | | 0.08 | ( | -0.24 | - | 0.40 | ) | 0.6140 |
| Tumor size | | | | | | | | | | | | | | | | | | | | | | | |
| ≤2cm | reference | | | | | | | | reference | | | | | | | | reference | | | | | | |
| >2cm | 0.00 | ( | -0.29 | - | 0.30 | ) | 0.9854 | | -0.05 | ( | -0.30 | - | 0.19 | ) | 0.6733 | | -0.16 | ( | -0.55 | - | 0.22 | ) | 0.4061 |
| PR status | | | | | | | | | | | | | | | | | | | | | | | |
| PR- | reference | | | | | | | | reference | | | | | | | | reference | | | | | | |
| PR+ | -0.06 | ( | -0.31 | - | 0.19 | ) | 0.6629 | | 0.01 | ( | -0.21 | - | 0.22 | ) | 0.9624 | | -0.17 | ( | -0.50 | - | 0.17 | ) | 0.3288 |
| HER2 status | | | | | | | | | | | | | | | | | | | | | | | |
| HER2- | reference | | | | | | | | reference | | | | | | | | reference | | | | | | |
| HER2+ | 0.27 | ( | -0.18 | - | 0.73 | ) | 0.2343 | | 0.27 | ( | -0.10 | - | 0.64 | ) | 0.1577 | | -0.01 | ( | -0.58 | - | 0.56 | ) | 0.9619 |
| PIK3CA status3 | | | | | | | | | | | | | | | | | | | | | | | |
| wild-type | reference | | | | | | | | reference | | | | | | | | reference | | | | | | |
| mutated | 0.15 | ( | -0.12 | - | 0.41 | ) | 0.2841 | | 0.20 | ( | -0.03 | - | 0.42 | ) | 0.0832 | | 0.15 | ( | -0.19 | - | 0.50 | ) | 0.3855 |
| | | | | | | | | | | | | | | | | | | | | | | | |
| | | | | | | | | | | | | | | | | | | | | | | | |
| (1) coefficient calculated by linear multivariable regression model; (2) four levels of p-AKT (Ser473) positivity was evaluated by pathologist according the cytoplasmatic intensity of the staining on tumor cells; (3) PIK3CA status based on hot-spot mutations in exon 9 (E542K and E545K) and exon 20 (H1047L and H1047R). Abbreviations: number of tumors included in each model (n), coefficient (Coeff.), 95% confidence interval (95%CI), progesterone receptor (PR). | | | | | | | | | | | | | | | | | | | | | | | |

## Slide 6
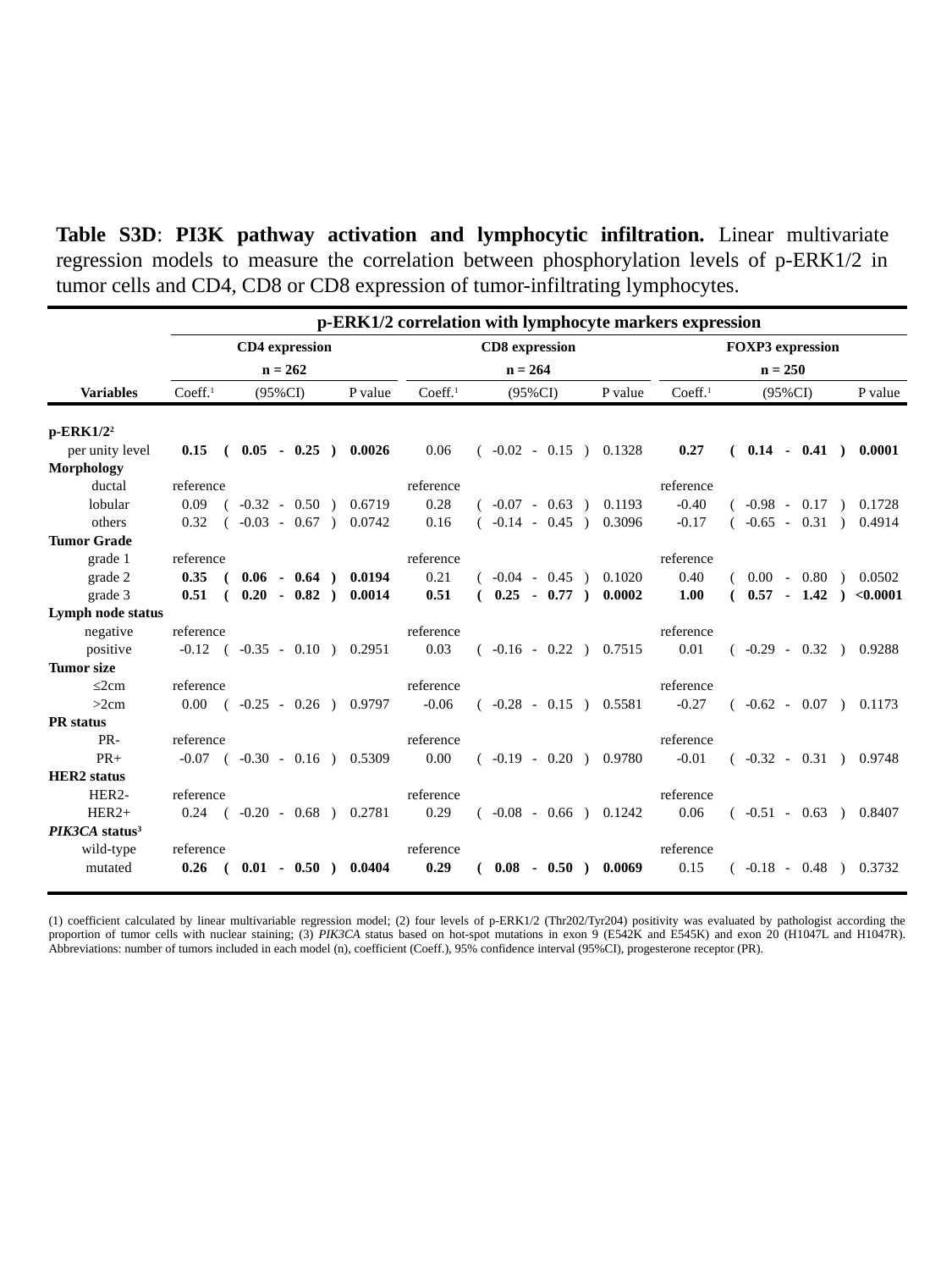

Table S3D: PI3K pathway activation and lymphocytic infiltration. Linear multivariate regression models to measure the correlation between phosphorylation levels of p-ERK1/2 in tumor cells and CD4, CD8 or CD8 expression of tumor-infiltrating lymphocytes.
| | p-ERK1/2 correlation with lymphocyte markers expression | | | | | | | | | | | | | | | | | | | | | | |
| --- | --- | --- | --- | --- | --- | --- | --- | --- | --- | --- | --- | --- | --- | --- | --- | --- | --- | --- | --- | --- | --- | --- | --- |
| | CD4 expression | | | | | | | | CD8 expression | | | | | | | | FOXP3 expression | | | | | | |
| | n = 262 | | | | | | | | n = 264 | | | | | | | | n = 250 | | | | | | |
| Variables | Coeff.1 | | (95%CI) | | | | P value | | Coeff.1 | | (95%CI) | | | | P value | | Coeff.1 | | (95%CI) | | | | P value |
| | | | | | | | | | | | | | | | | | | | | | | | |
| p-ERK1/22 | | | | | | | | | | | | | | | | | | | | | | | |
| per unity level | 0.15 | ( | 0.05 | - | 0.25 | ) | 0.0026 | | 0.06 | ( | -0.02 | - | 0.15 | ) | 0.1328 | | 0.27 | ( | 0.14 | - | 0.41 | ) | 0.0001 |
| Morphology | | | | | | | | | | | | | | | | | | | | | | | |
| ductal | reference | | | | | | | | reference | | | | | | | | reference | | | | | | |
| lobular | 0.09 | ( | -0.32 | - | 0.50 | ) | 0.6719 | | 0.28 | ( | -0.07 | - | 0.63 | ) | 0.1193 | | -0.40 | ( | -0.98 | - | 0.17 | ) | 0.1728 |
| others | 0.32 | ( | -0.03 | - | 0.67 | ) | 0.0742 | | 0.16 | ( | -0.14 | - | 0.45 | ) | 0.3096 | | -0.17 | ( | -0.65 | - | 0.31 | ) | 0.4914 |
| Tumor Grade | | | | | | | | | | | | | | | | | | | | | | | |
| grade 1 | reference | | | | | | | | reference | | | | | | | | reference | | | | | | |
| grade 2 | 0.35 | ( | 0.06 | - | 0.64 | ) | 0.0194 | | 0.21 | ( | -0.04 | - | 0.45 | ) | 0.1020 | | 0.40 | ( | 0.00 | - | 0.80 | ) | 0.0502 |
| grade 3 | 0.51 | ( | 0.20 | - | 0.82 | ) | 0.0014 | | 0.51 | ( | 0.25 | - | 0.77 | ) | 0.0002 | | 1.00 | ( | 0.57 | - | 1.42 | ) | <0.0001 |
| Lymph node status | | | | | | | | | | | | | | | | | | | | | | | |
| negative | reference | | | | | | | | reference | | | | | | | | reference | | | | | | |
| positive | -0.12 | ( | -0.35 | - | 0.10 | ) | 0.2951 | | 0.03 | ( | -0.16 | - | 0.22 | ) | 0.7515 | | 0.01 | ( | -0.29 | - | 0.32 | ) | 0.9288 |
| Tumor size | | | | | | | | | | | | | | | | | | | | | | | |
| ≤2cm | reference | | | | | | | | reference | | | | | | | | reference | | | | | | |
| >2cm | 0.00 | ( | -0.25 | - | 0.26 | ) | 0.9797 | | -0.06 | ( | -0.28 | - | 0.15 | ) | 0.5581 | | -0.27 | ( | -0.62 | - | 0.07 | ) | 0.1173 |
| PR status | | | | | | | | | | | | | | | | | | | | | | | |
| PR- | reference | | | | | | | | reference | | | | | | | | reference | | | | | | |
| PR+ | -0.07 | ( | -0.30 | - | 0.16 | ) | 0.5309 | | 0.00 | ( | -0.19 | - | 0.20 | ) | 0.9780 | | -0.01 | ( | -0.32 | - | 0.31 | ) | 0.9748 |
| HER2 status | | | | | | | | | | | | | | | | | | | | | | | |
| HER2- | reference | | | | | | | | reference | | | | | | | | reference | | | | | | |
| HER2+ | 0.24 | ( | -0.20 | - | 0.68 | ) | 0.2781 | | 0.29 | ( | -0.08 | - | 0.66 | ) | 0.1242 | | 0.06 | ( | -0.51 | - | 0.63 | ) | 0.8407 |
| PIK3CA status3 | | | | | | | | | | | | | | | | | | | | | | | |
| wild-type | reference | | | | | | | | reference | | | | | | | | reference | | | | | | |
| mutated | 0.26 | ( | 0.01 | - | 0.50 | ) | 0.0404 | | 0.29 | ( | 0.08 | - | 0.50 | ) | 0.0069 | | 0.15 | ( | -0.18 | - | 0.48 | ) | 0.3732 |
| | | | | | | | | | | | | | | | | | | | | | | | |
| | | | | | | | | | | | | | | | | | | | | | | | |
| (1) coefficient calculated by linear multivariable regression model; (2) four levels of p-ERK1/2 (Thr202/Tyr204) positivity was evaluated by pathologist according the proportion of tumor cells with nuclear staining; (3) PIK3CA status based on hot-spot mutations in exon 9 (E542K and E545K) and exon 20 (H1047L and H1047R). Abbreviations: number of tumors included in each model (n), coefficient (Coeff.), 95% confidence interval (95%CI), progesterone receptor (PR). | | | | | | | | | | | | | | | | | | | | | | | |

## Slide 7
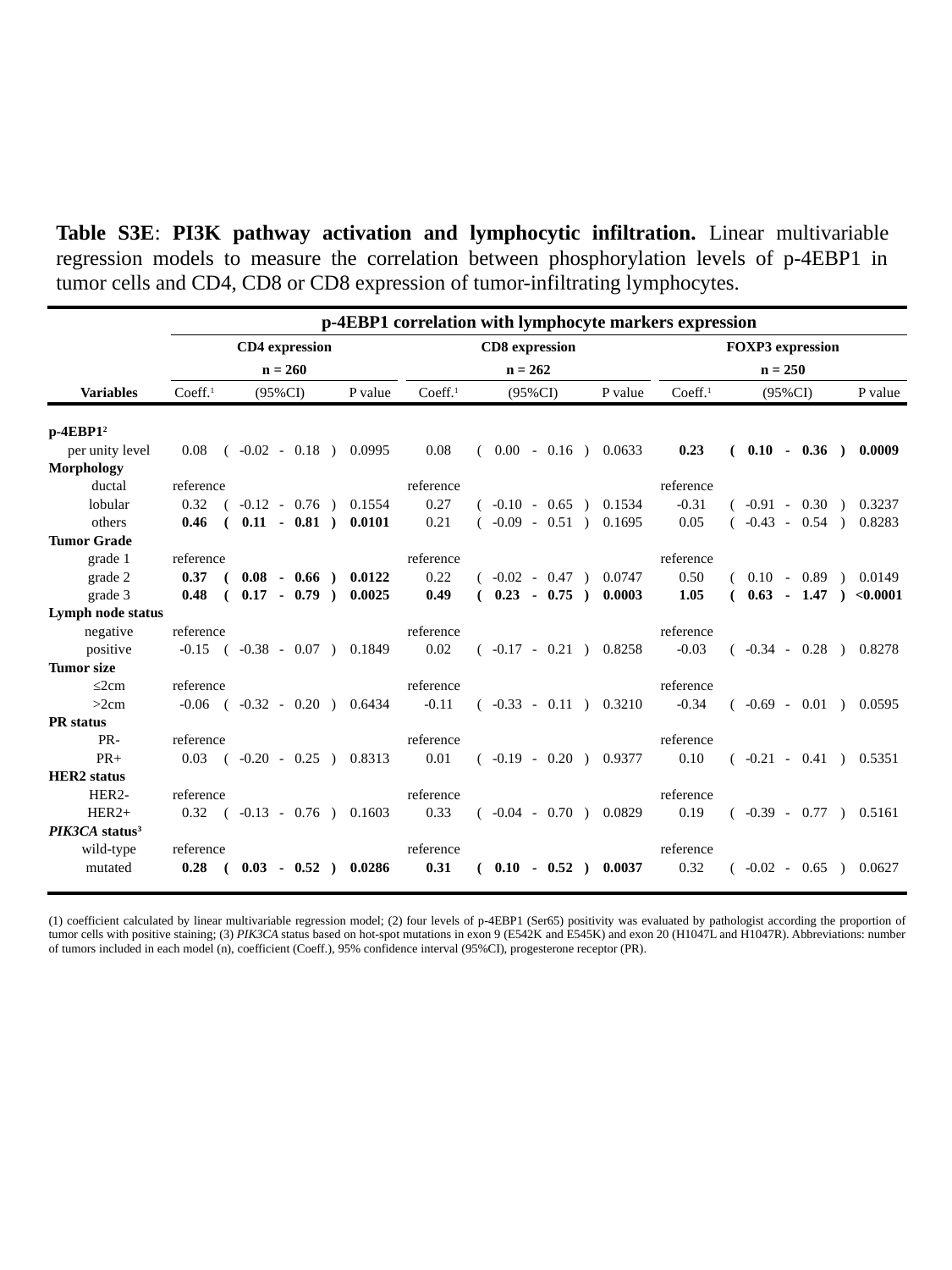

Table S3E: PI3K pathway activation and lymphocytic infiltration. Linear multivariable regression models to measure the correlation between phosphorylation levels of p-4EBP1 in tumor cells and CD4, CD8 or CD8 expression of tumor-infiltrating lymphocytes.
| | p-4EBP1 correlation with lymphocyte markers expression | | | | | | | | | | | | | | | | | | | | | | |
| --- | --- | --- | --- | --- | --- | --- | --- | --- | --- | --- | --- | --- | --- | --- | --- | --- | --- | --- | --- | --- | --- | --- | --- |
| | CD4 expression | | | | | | | | CD8 expression | | | | | | | | FOXP3 expression | | | | | | |
| | n = 260 | | | | | | | | n = 262 | | | | | | | | n = 250 | | | | | | |
| Variables | Coeff.1 | | (95%CI) | | | | P value | | Coeff.1 | | (95%CI) | | | | P value | | Coeff.1 | | (95%CI) | | | | P value |
| | | | | | | | | | | | | | | | | | | | | | | | |
| p-4EBP12 | | | | | | | | | | | | | | | | | | | | | | | |
| per unity level | 0.08 | ( | -0.02 | - | 0.18 | ) | 0.0995 | | 0.08 | ( | 0.00 | - | 0.16 | ) | 0.0633 | | 0.23 | ( | 0.10 | - | 0.36 | ) | 0.0009 |
| Morphology | | | | | | | | | | | | | | | | | | | | | | | |
| ductal | reference | | | | | | | | reference | | | | | | | | reference | | | | | | |
| lobular | 0.32 | ( | -0.12 | - | 0.76 | ) | 0.1554 | | 0.27 | ( | -0.10 | - | 0.65 | ) | 0.1534 | | -0.31 | ( | -0.91 | - | 0.30 | ) | 0.3237 |
| others | 0.46 | ( | 0.11 | - | 0.81 | ) | 0.0101 | | 0.21 | ( | -0.09 | - | 0.51 | ) | 0.1695 | | 0.05 | ( | -0.43 | - | 0.54 | ) | 0.8283 |
| Tumor Grade | | | | | | | | | | | | | | | | | | | | | | | |
| grade 1 | reference | | | | | | | | reference | | | | | | | | reference | | | | | | |
| grade 2 | 0.37 | ( | 0.08 | - | 0.66 | ) | 0.0122 | | 0.22 | ( | -0.02 | - | 0.47 | ) | 0.0747 | | 0.50 | ( | 0.10 | - | 0.89 | ) | 0.0149 |
| grade 3 | 0.48 | ( | 0.17 | - | 0.79 | ) | 0.0025 | | 0.49 | ( | 0.23 | - | 0.75 | ) | 0.0003 | | 1.05 | ( | 0.63 | - | 1.47 | ) | <0.0001 |
| Lymph node status | | | | | | | | | | | | | | | | | | | | | | | |
| negative | reference | | | | | | | | reference | | | | | | | | reference | | | | | | |
| positive | -0.15 | ( | -0.38 | - | 0.07 | ) | 0.1849 | | 0.02 | ( | -0.17 | - | 0.21 | ) | 0.8258 | | -0.03 | ( | -0.34 | - | 0.28 | ) | 0.8278 |
| Tumor size | | | | | | | | | | | | | | | | | | | | | | | |
| ≤2cm | reference | | | | | | | | reference | | | | | | | | reference | | | | | | |
| >2cm | -0.06 | ( | -0.32 | - | 0.20 | ) | 0.6434 | | -0.11 | ( | -0.33 | - | 0.11 | ) | 0.3210 | | -0.34 | ( | -0.69 | - | 0.01 | ) | 0.0595 |
| PR status | | | | | | | | | | | | | | | | | | | | | | | |
| PR- | reference | | | | | | | | reference | | | | | | | | reference | | | | | | |
| PR+ | 0.03 | ( | -0.20 | - | 0.25 | ) | 0.8313 | | 0.01 | ( | -0.19 | - | 0.20 | ) | 0.9377 | | 0.10 | ( | -0.21 | - | 0.41 | ) | 0.5351 |
| HER2 status | | | | | | | | | | | | | | | | | | | | | | | |
| HER2- | reference | | | | | | | | reference | | | | | | | | reference | | | | | | |
| HER2+ | 0.32 | ( | -0.13 | - | 0.76 | ) | 0.1603 | | 0.33 | ( | -0.04 | - | 0.70 | ) | 0.0829 | | 0.19 | ( | -0.39 | - | 0.77 | ) | 0.5161 |
| PIK3CA status3 | | | | | | | | | | | | | | | | | | | | | | | |
| wild-type | reference | | | | | | | | reference | | | | | | | | reference | | | | | | |
| mutated | 0.28 | ( | 0.03 | - | 0.52 | ) | 0.0286 | | 0.31 | ( | 0.10 | - | 0.52 | ) | 0.0037 | | 0.32 | ( | -0.02 | - | 0.65 | ) | 0.0627 |
| | | | | | | | | | | | | | | | | | | | | | | | |
| | | | | | | | | | | | | | | | | | | | | | | | |
| (1) coefficient calculated by linear multivariable regression model; (2) four levels of p-4EBP1 (Ser65) positivity was evaluated by pathologist according the proportion of tumor cells with positive staining; (3) PIK3CA status based on hot-spot mutations in exon 9 (E542K and E545K) and exon 20 (H1047L and H1047R). Abbreviations: number of tumors included in each model (n), coefficient (Coeff.), 95% confidence interval (95%CI), progesterone receptor (PR). | | | | | | | | | | | | | | | | | | | | | | | |

## Slide 8
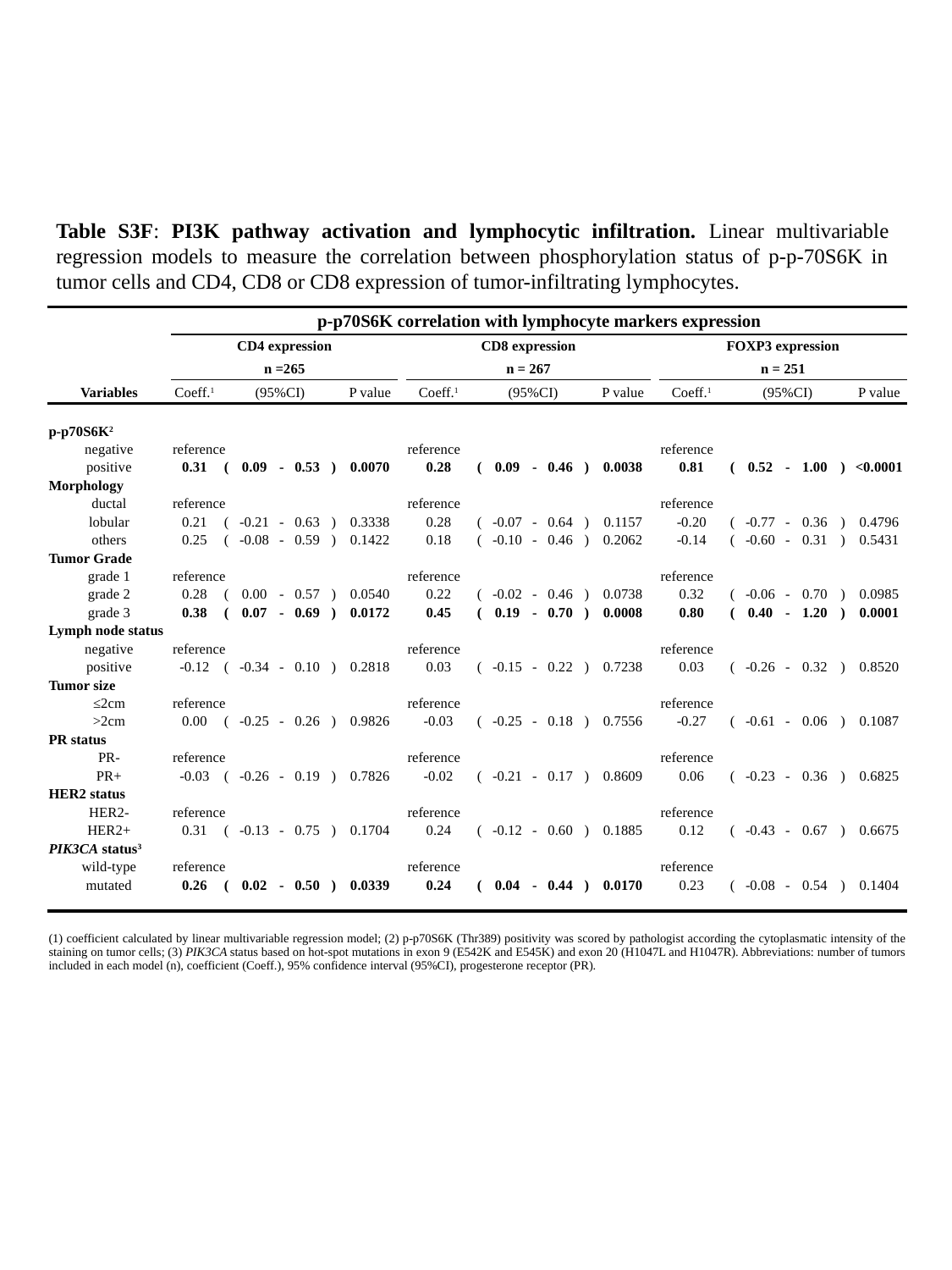

Table S3F: PI3K pathway activation and lymphocytic infiltration. Linear multivariable regression models to measure the correlation between phosphorylation status of p-p-70S6K in tumor cells and CD4, CD8 or CD8 expression of tumor-infiltrating lymphocytes.
| | p-p70S6K correlation with lymphocyte markers expression | | | | | | | | | | | | | | | | | | | | | | |
| --- | --- | --- | --- | --- | --- | --- | --- | --- | --- | --- | --- | --- | --- | --- | --- | --- | --- | --- | --- | --- | --- | --- | --- |
| | CD4 expression | | | | | | | | CD8 expression | | | | | | | | FOXP3 expression | | | | | | |
| | n =265 | | | | | | | | n = 267 | | | | | | | | n = 251 | | | | | | |
| Variables | Coeff.1 | | (95%CI) | | | | P value | | Coeff.1 | | (95%CI) | | | | P value | | Coeff.1 | | (95%CI) | | | | P value |
| | | | | | | | | | | | | | | | | | | | | | | | |
| p-p70S6K2 | | | | | | | | | | | | | | | | | | | | | | | |
| negative | reference | | | | | | | | reference | | | | | | | | reference | | | | | | |
| positive | 0.31 | ( | 0.09 | - | 0.53 | ) | 0.0070 | | 0.28 | ( | 0.09 | - | 0.46 | ) | 0.0038 | | 0.81 | ( | 0.52 | - | 1.00 | ) | <0.0001 |
| Morphology | | | | | | | | | | | | | | | | | | | | | | | |
| ductal | reference | | | | | | | | reference | | | | | | | | reference | | | | | | |
| lobular | 0.21 | ( | -0.21 | - | 0.63 | ) | 0.3338 | | 0.28 | ( | -0.07 | - | 0.64 | ) | 0.1157 | | -0.20 | ( | -0.77 | - | 0.36 | ) | 0.4796 |
| others | 0.25 | ( | -0.08 | - | 0.59 | ) | 0.1422 | | 0.18 | ( | -0.10 | - | 0.46 | ) | 0.2062 | | -0.14 | ( | -0.60 | - | 0.31 | ) | 0.5431 |
| Tumor Grade | | | | | | | | | | | | | | | | | | | | | | | |
| grade 1 | reference | | | | | | | | reference | | | | | | | | reference | | | | | | |
| grade 2 | 0.28 | ( | 0.00 | - | 0.57 | ) | 0.0540 | | 0.22 | ( | -0.02 | - | 0.46 | ) | 0.0738 | | 0.32 | ( | -0.06 | - | 0.70 | ) | 0.0985 |
| grade 3 | 0.38 | ( | 0.07 | - | 0.69 | ) | 0.0172 | | 0.45 | ( | 0.19 | - | 0.70 | ) | 0.0008 | | 0.80 | ( | 0.40 | - | 1.20 | ) | 0.0001 |
| Lymph node status | | | | | | | | | | | | | | | | | | | | | | | |
| negative | reference | | | | | | | | reference | | | | | | | | reference | | | | | | |
| positive | -0.12 | ( | -0.34 | - | 0.10 | ) | 0.2818 | | 0.03 | ( | -0.15 | - | 0.22 | ) | 0.7238 | | 0.03 | ( | -0.26 | - | 0.32 | ) | 0.8520 |
| Tumor size | | | | | | | | | | | | | | | | | | | | | | | |
| ≤2cm | reference | | | | | | | | reference | | | | | | | | reference | | | | | | |
| >2cm | 0.00 | ( | -0.25 | - | 0.26 | ) | 0.9826 | | -0.03 | ( | -0.25 | - | 0.18 | ) | 0.7556 | | -0.27 | ( | -0.61 | - | 0.06 | ) | 0.1087 |
| PR status | | | | | | | | | | | | | | | | | | | | | | | |
| PR- | reference | | | | | | | | reference | | | | | | | | reference | | | | | | |
| PR+ | -0.03 | ( | -0.26 | - | 0.19 | ) | 0.7826 | | -0.02 | ( | -0.21 | - | 0.17 | ) | 0.8609 | | 0.06 | ( | -0.23 | - | 0.36 | ) | 0.6825 |
| HER2 status | | | | | | | | | | | | | | | | | | | | | | | |
| HER2- | reference | | | | | | | | reference | | | | | | | | reference | | | | | | |
| HER2+ | 0.31 | ( | -0.13 | - | 0.75 | ) | 0.1704 | | 0.24 | ( | -0.12 | - | 0.60 | ) | 0.1885 | | 0.12 | ( | -0.43 | - | 0.67 | ) | 0.6675 |
| PIK3CA status3 | | | | | | | | | | | | | | | | | | | | | | | |
| wild-type | reference | | | | | | | | reference | | | | | | | | reference | | | | | | |
| mutated | 0.26 | ( | 0.02 | - | 0.50 | ) | 0.0339 | | 0.24 | ( | 0.04 | - | 0.44 | ) | 0.0170 | | 0.23 | ( | -0.08 | - | 0.54 | ) | 0.1404 |
| | | | | | | | | | | | | | | | | | | | | | | | |
| | | | | | | | | | | | | | | | | | | | | | | | |
| (1) coefficient calculated by linear multivariable regression model; (2) p-p70S6K (Thr389) positivity was scored by pathologist according the cytoplasmatic intensity of the staining on tumor cells; (3) PIK3CA status based on hot-spot mutations in exon 9 (E542K and E545K) and exon 20 (H1047L and H1047R). Abbreviations: number of tumors included in each model (n), coefficient (Coeff.), 95% confidence interval (95%CI), progesterone receptor (PR). | | | | | | | | | | | | | | | | | | | | | | | |

## Slide 9
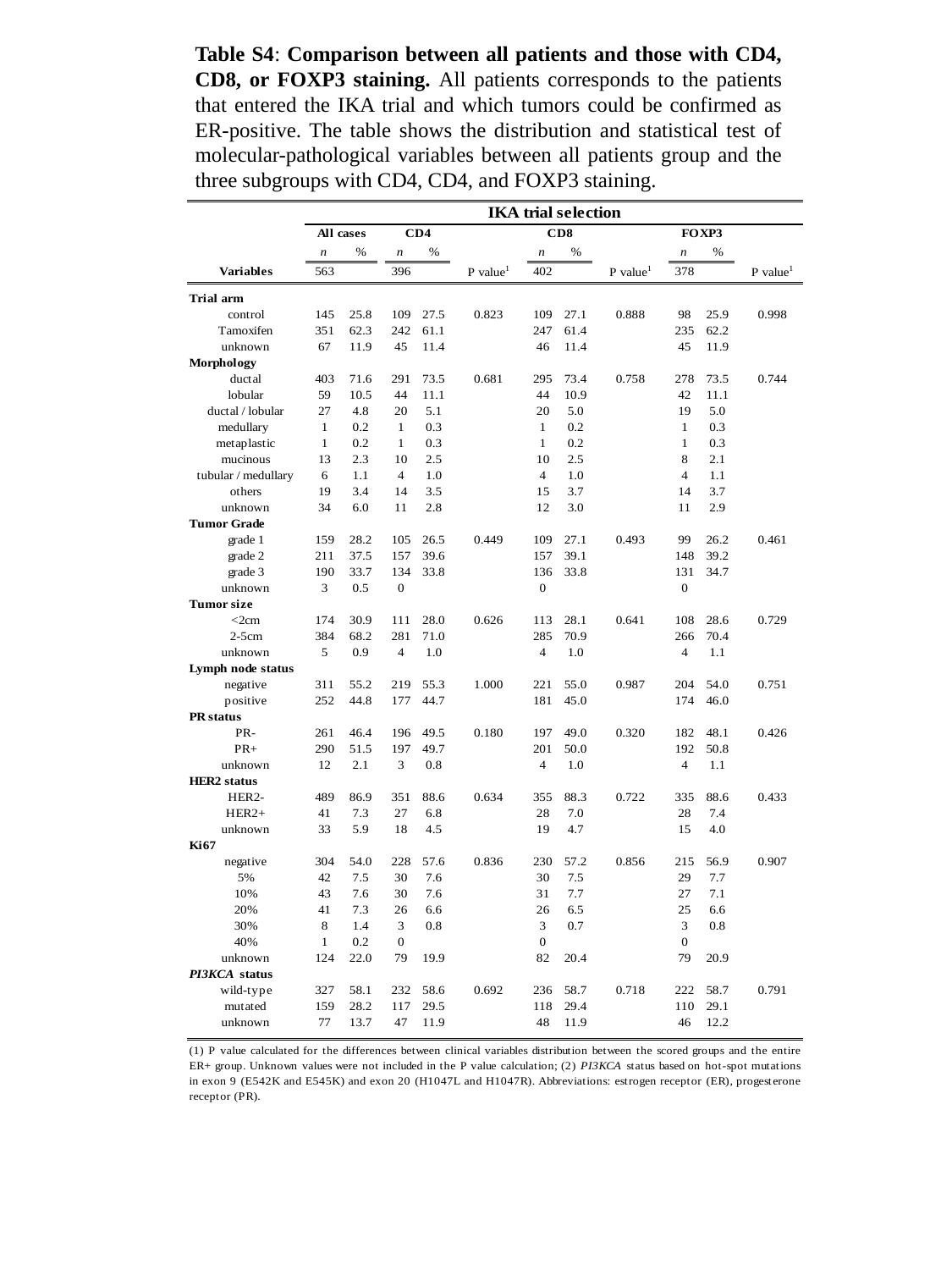

Table S4: Comparison between all patients and those with CD4, CD8, or FOXP3 staining. All patients corresponds to the patients that entered the IKA trial and which tumors could be confirmed as ER-positive. The table shows the distribution and statistical test of molecular-pathological variables between all patients group and the three subgroups with CD4, CD4, and FOXP3 staining.
